# Magnetic Adsorbents for Wastewater Treatment: Advancements in Their Synthesis Methods

**DOI:** 10.3390/ma15031053

**Published:** 2022-01-29

**Authors:** Vanpaseuth Phouthavong, Ruixin Yan, Supinya Nijpanich, Takeshi Hagio, Ryoichi Ichino, Long Kong, Liang Li

**Affiliations:** 1 Department of Chemical Systems Engineering, Graduate School of Engineering, Nagoya University, Furo-cho, Chikusa-ku, Nagoya 464-8603, Japan; phouthavong.vanpaseuth@i.mbox.nagoya-u.ac.jp (V.P.); supinya@slri.or.th (S.N.); hagio@mirai.nagoya-u.ac.jp (T.H.); 2 School of Environmental Science and Engineering, Shanghai Jiao Tong University, Shanghai 200240, China; risonforever2018@gmail.com (R.Y.); liangli117@sjtu.edu.cn (L.L.); 3 Institute of Materials Innovation, Institutes for Innovation for Future Society, Nagoya University, Furo-cho, Chikusa-ku, Nagoya 464-8601, Japan

**Keywords:** magnetic adsorbent, synthesis methods, material structure, water treatment

## Abstract

The remediation of water streams, polluted by various substances, is important for realizing a sustainable future. Magnetic adsorbents are promising materials for wastewater treatment. Although numerous techniques have been developed for the preparation of magnetic adsorbents, with effective adsorption performance, reviews that focus on the synthesis methods of magnetic adsorbents for wastewater treatment and their material structures have not been reported. In this review, advancements in the synthesis methods of magnetic adsorbents for the removal of substances from water streams has been comprehensively summarized and discussed. Generally, the synthesis methods are categorized into five groups, as follows: direct use of magnetic particles as adsorbents, attachment of pre-prepared adsorbents and pre-prepared magnetic particles, synthesis of magnetic particles on pre-prepared adsorbents, synthesis of adsorbents on preprepared magnetic particles, and co-synthesis of adsorbents and magnetic particles. The main improvements in the advanced methods involved making the conventional synthesis a less energy intensive, more efficient, and simpler process, while maintaining or increasing the adsorption performance. The key challenges, such as the enhancement of the adsorption performance of materials and the design of sophisticated material structures, are discussed as well.

## 1. Introduction

### 1.1. Importance and Advantage of Magnetic Adsorbent Technologies for Wastewater Treatment

Natural water sources, apart from soil and air, are the primary medium for pollutant mobility. Over the decades, the release of untreated wastewater into natural water sources, coupled with an ever-increasing population, has resulted in severe environmental problems. Enormous quantities of chemicals, such as pesticides, antibiotics, heavy metals, and dyes are used each year, to drive the global economy. As clean water sources are limited, wastewater should be recycled, by utilizing effective chemical, physical, and biological treatment technologies. Among these techniques, adsorption is a widely exploited physical method because it is simple, environmentally friendly, cost-effective, and reusable [1]. Adsorbents used for the removal of pollutants can be developed to selectively adsorb target adsorbates, with high adsorption performance. The collection of adsorbents after adsorption should be quick and easy. The magnetic separation technique is a good option because magnetic materials can be separated easily from mixtures by applying a magnetic field. The first application of magnetic separation [2] was reported by Robinson et al. [3], in 1973, for the separation of enzymes immobilized on the surface of iron oxide particles. Since then, the application of magnetic separation in adsorption has been extended in different ways, described below:

(i) Addition of magnetic particles (iron (Fe) or iron oxides (Fe_x_O_y_)) to assist in the separation of precipitates; that is, coagulation–flocculation during the water treatment process [4,5];
(ii) Direct use of magnetic materials as adsorbents [6];
(iii) Combining magnetic particles with adsorbents to yield magnetic adsorbents [7].

**Figure 1:**
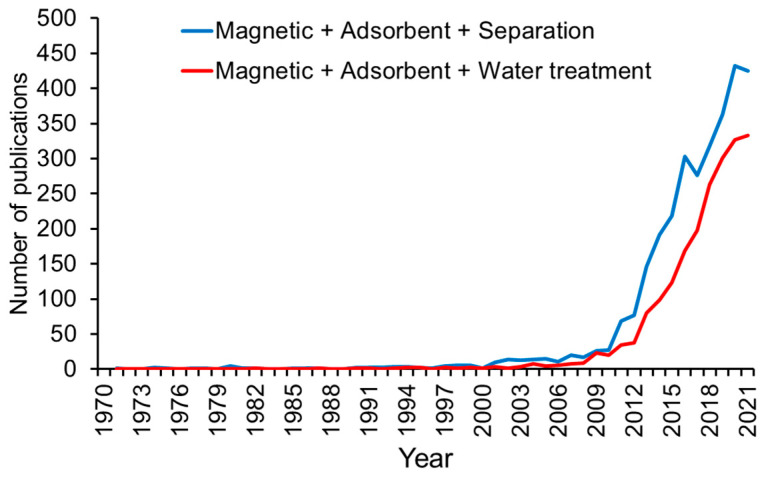
Number of publications that contain the keywords “magnetic + adsorbent + separation” and “magnetic + adsorbent + water treatment” in Scopus. (Accessed on 2 January 2022).

The latter two alternatives have received more interest. Keyword searches in Scopus for “magnetic + adsorbent + separation” and “magnetic + adsorbent + water treatment”, reveal a growing trend in the number of research articles (Figure 1).

### 1.2. History and Advancements of Magnetic Adsorbents

Iron-based materials are typically used as magnetic particles because of the strong magnetic moment of unpaired electrons in the 3D orbitals of Fe atoms. The most commonly used materials in the adsorption process are magnetic materials, such as Fe, magnetite (Fe_3_O_4_), and maghemite (γ-Fe_2_O_3_). Though Fe and Fe_3_O_4_ exhibit strong responses to magnetic fields, Fe_3_O_4_ is used most widely in the literature, owing to its higher stability. Fe_3_O_4_ particles are superparamagnetic at a size of several nanometers [8]. In aqueous solutions, the abundant hydroxyl groups on the surface of Fe_3_O_4_ can be protonated or deprotonated by varying the pH, to generate positive or negative charges. Therefore, Fe_3_O_4_ can be used as an adsorbent to remove ionic species from water via electrostatic interactions [9]. The Fe atom in Fe_3_O_4_ also acts as an adsorption site for complexation with negatively charged species or the electron-rich functional groups of some organic pollutants. Further, the surface of Fe_3_O_4_ can be modified to improve functionalization and protect the magnetic core from magnetism degradation [10]. Magnetic adsorbents offer the following advantages:

(i) They can be easily separated from bulk solutions by applying an external magnetic field;
(ii) They are environmentally friendly owing to their biocompatibility [11];
(iii) They are reusable;
(iv) Various organic and inorganic functional groups, to remove diverse target pollutants, can be prepared and modified at the laboratory scale.

In addition to environmental remediation applications, magnetic adsorbents have been used in analytical chemistry in the so-called magnetic solid-phase extraction sample preparation technique. This technique is used to clean up and pre-concentrate the samples, before core analysis improves the detection of anions [12] and organic pollutants [13].

Although magnetic iron oxides are naturally occurring, we can control particle size and achieve better purity by synthesizing them in the laboratory via various routes, such as co-precipitation [14], hydrothermal [15], solvothermal [16], thermal decomposition [17], microemulsion [18], electrochemical [19], and microwave-assisted [20] methods, and greener synthesis methods using biogenic materials [21,22]. Functionalized magnetic particles, or magnetic composites, have been developed or modified using these methods. Among these methods, co-precipitation is the most widely used, due to its simple operation at low temperatures. However, the affinity of hydroxy groups on conventional magnetic iron oxides limits the adsorption performance of some target cationic species. Consequently, they have been coupled with functional adsorbents, such as polymeric ion-exchange resins, used in water treatment processes [23,24]. The magnetic polymer, prepared by the polymerization of γ-Fe_2_O_3_, is useful for water softening, desalinization, and oil removal from water. In 1996, Chen et al. [25] prepared magnetic composites by simply precipitating ferrihydrite (Fe(OH)_3_) from Fe_3_O_4_ and used it to remove heavy metals from water. Since then, the synthesis of magnetic adsorbents has drawn considerable attention, as indicated by the growing number of publications. Diverse effective adsorbents have been reported to incorporate magnetic particles, such as metal oxides [26,27], silica-based materials [28,29,30,31,32], carbon-based materials [33,34,35], graphene oxide [36], biopolymers, such as lignin [37], cellulose [38], chitosan [39], synthetic polymers [40,41,42], molecularly imprinted polymers [43], metal organic frameworks [44], and biowaste [45,46]. Magnetic adsorbents can be prepared using physical methods, such as mechanical agitation (stirring, vibration, milling, and ultrasonication) at room or elevated temperatures in some cases, along with the addition of adhesives. Examples of these physical attachments are discussed concretely in this review. Although magnetic adsorbents prepared by physical methods show reasonable adsorption performance, the loose attachment results in a deterioration of their morphology. A low adsorption performance could be due to hidden adsorption sites attaching to the magnetic particles. In contrast, chemically bonded magnetic adsorbents can overcome these drawbacks. Conventional chemical methods utilize specific chemical reactions to form magnetic particles, on pre-synthesized adsorbents or adsorbents on magnetic particles under certain treatment conditions, such as hydrothermal, solvothermal, or reflux.

The adsorption performance of Fe_3_O_4_ can be improved by doping with sulfur [47,48]. This technology has also been applied to composite adsorbents; namely, converting the Fe_3_O_4_ priorly embedded onto adsorbents to Fe_3_S_4_ [49]. The challenge in magnetic adsorbent synthesis is not only to improve the adsorption performance, but also to accomplish good distribution or incorporation of magnetic particles in the adsorbent morphology. Advanced synthesis routes, such as the seed-assisted method [29], pre-milling of magnetic particles with adsorbent seed crystals [30], embedding magnetic particles with a carbon source before carbonization [34], or pre-attaching magnetic particles with precursors as a dry gel before heat treatment [31], help to improve the morphology of magnetic adsorbents. Furthermore, challenges to co-synthesizing magnetic particles and adsorbents in a one-pot synthesis have recently been explored [26].

### 1.3. Previous Reviews

Many review papers have been published about magnetic particles (not magnetic adsorbents), based on different perspectives, such as the type of surface coating materials, phase of synthesis media, type of reactor, and potential applications. In the area of catalysts and medicine, Lu et al. [10] and Wu et al. [8] classified the synthesis methods of magnetic materials into four popular methods; namely, co-precipitation, thermal decomposition, microemulsion, and hydrothermal synthesis, and also reviewed the surface modification of magnetic particles, based on the type of coating materials. In addition, Teja et al. [50] summarized the synthesis methods of iron oxides and categorized them, according to phase of synthesis media, such as gas phase, liquid phase, and two-phase methods, for the same scientific area. Synthesis methods of magnetic particles, sub-divided using more specific terminology, were found in some review articles [51,52]. Akbarzadeh et al. [53] focused on magnetic particles for biomedicines. Ali et al. [52] comprehensively summarized and discussed various applications for magnetic materials, for industrial, biomedical, environmental, agricultural, and analytical purposes. Ali’s group introduced applications for wastewater treatment briefly, in the section for environmental application. Recently, García-Merino et al. [54] reviewed the synthesis of magnetic particles based on types of reactors. They summarized and compared features of conventional batch synthesis with continuous microfluidic synthesis methods. It was found that most of the above reviews focus on the classification of treatment processes, such as physical, chemical, or biological methods. The relationship between synthesis methods and performance in wastewater treatment has seldom been studied.

As for magnetic adsorbents, Reshadi et al. [55] reviewed magnetic adsorbents used for landfill leachate treatment. Furthermore, magnetic adsorbents for wastewater treatment have been reported by Shukla et al. [56], from the viewpoint of the synthesis methods of magnetic nanoparticles. Abdullah et al. [57] summarized research articles, regarding the synthesis of magnetic adsorbents that used conventional methods, but mainly focused on various types of solid substrates, which were common adsorbents, such as silica-based materials, carbon materials, and graphene, to embed the magnetic particles. Apart from synthesis methods, magnetic adsorbents have been reviewed from another point of view. Abdel Maksoud et al. [58] presented the advantages of using magnetic adsorbents, by classifying them based on types of applied magnetic materials. They additionally discussed their properties and removal efficiency towards organic and inorganic pollutants.

### 1.4. Objective of This Review

As summarized above, numerous techniques have been developed for the preparation of magnetic adsorbents with effective adsorption performance. To date, magnetic adsorbents have been reviewed from different perspectives; however, reviews that focus on the synthesis methods and the obtained material structures have not been reported. In this review, magnetic adsorbents, mostly used for water pollutant removal, are reviewed and classified by focusing on the synthesis method and the attaching force between magnetic materials and adsorbents, within the obtained material structure. The advantages and drawbacks of these methods, together with the magnetic properties and adsorption performance of the materials, are discussed. Additionally, research papers that illustrate the advancement of each category are summarized.

## 2. Conventional Synthesis Methods of Magnetic Adsorbents

Conventional synthesis methods can be categorized into four primary groups. Conventional magnetic adsorbents used for the removal of various organic and inorganic pollutants from water, according to their groups with their synthesis methods, adsorption performances, and magnetic performances, are summarized in Table 1.

### 2.1. Adsorption Using Magnetic Material Adsorbents

Various magnetic materials, especially iron-based materials, such as nano zerovalent iron (nZVI), maghemite (γ-Fe_2_O_3_), and magnetite (Fe_3_O_4_), have received extensive attention for use as magnetic adsorbents. The effective control of particle sizes, crystal structures, and shapes are key issues in the synthesis of magnetic material adsorbents.

#### 2.1.1. nZVI

A wide range of experimental studies have been conducted on magnetic nZVI, owing to its strong reducing properties. Through surface corrosion and precipitation, nZVI can efficiently reduce heavy metal ions, such as hexavalent chromium (Cr(VI)) and some pollutants with low degradability. Compared to other conventional decontamination methods, such as precipitation, ion exchange, and metal complexes, adsorption by nZVI has many advantages. For example, ZVI-based composites could be more cost-effective for in situ environmental applications, and the oxides or hydroxides of iron are natural minerals, which makes the composites eco-friendly.

Chemical reaction, due to its simplicity, is the most frequently used method to synthesize nZVI. Among chemical reactions, the use of sodium borohydride (NaBH_4_) to reduce the Fe-containing precursor at elevated temperatures has been very popular. Another chemical method to obtain nZVI is the reduction of ferrous oxides in an H_2_ atmosphere, at around 500 °C. In addition, some physical methods, such as ball milling and ultrasound assistance, wherein the particle size can be decreased from micrometer to nanometer without using any toxic reagents, can be used to obtain nZVI in a much easier and safer way.

**Figure 2:**
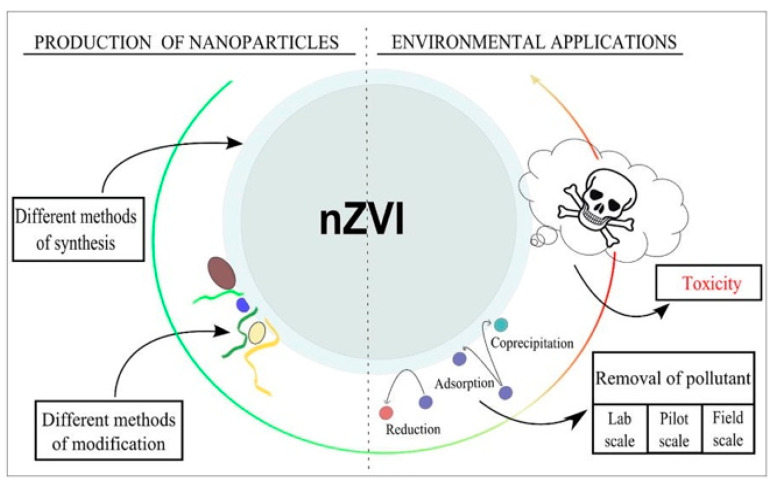
Brief depiction of the synthesis and environmental applications of nZVI (Reprinted from Chem. Eng. J., 287, Stefaniuk, M.; Oleszczuk, P.; Ok, Y.S. Review on nano zerovalent iron (nZVI): From synthesis to environmental applications, 618–632. Ref. [143], Copyright © 2022 with permission from Elsevier).

Bare nZVI particles have a large surface area, which is beneficial for increasing the removal capacity. The capacity reached 40 mg/g in a Cd(II) solution, though the capacity varied with different initial concentrations or different initial pH values [142]. However, surface oxidation is a serious problem that limits the further applications of nZVI; the powerful reducing property can act on non-target pollutants, decreasing the selectivity. In air, the formation of a passivation layer inhibits the reactivity of nZVI. Moreover, nZVI particles aggregate much more easily than other non-magnetic, iron-based materials, owing to the surface tension. Surface coating engineering and template confinement can help address these problems (Figure 2).

#### 2.1.2. γ-Fe_2_O_3_

γ-Fe_2_O_3_ is mainly obtained by the oxidation of iron, magnetite, or organometallic iron, or the calcination of iron hydroxide. As γ-Fe_2_O_3_ is a stable iron oxide, its adsorption to heavy metals can often be attributed to physical interactions, such as electrostatic attraction or van der Waals forces. Physical bonding has poor stability in preventing the adsorbents from leaching nanoparticle cores or metal pollutants. Therefore, the construction and design of adsorbents that form more chemical bonds have been investigated. Ahmed et al. [63] studied a mixture of Fe_3_O_4_ and γ-Fe_2_O_3_ particles, to remove Pb(II), Cr(III), and Cd(II); the removal capacity reached 617.3, 277.0, and 223.7 mg/g, respectively. For phosphate, this type of mixed adsorbent can also achieve a 95% removal efficiency [18]. Another mixed iron oxide, α-Fe_2_O_3_ and γ-Fe_2_O_3_, could uptake 46.5 mg/g As(III), by forming inner-sphere surface complexes [61].

The magnetism and particle sizes of iron oxide nanoparticles are affected by the preparation methods. Hydrothermal methods can achieve smaller nanoparticles and other methods may have aggregation problems. Hence, the stabilizer and surfactant materials should be added in the synthesis process to ensure an even dispersion of the particles. Moreover, the nature of chemical or physical bonding should be further understood, by investigating the mechanisms between the adsorbents and pollutants.

#### 2.1.3. Fe_3_O_4_

Magnetic Fe_3_O_4_ has been extensively studied because of its stability, natural abundance, low cost, and environmental friendliness.

**Figure 3:**
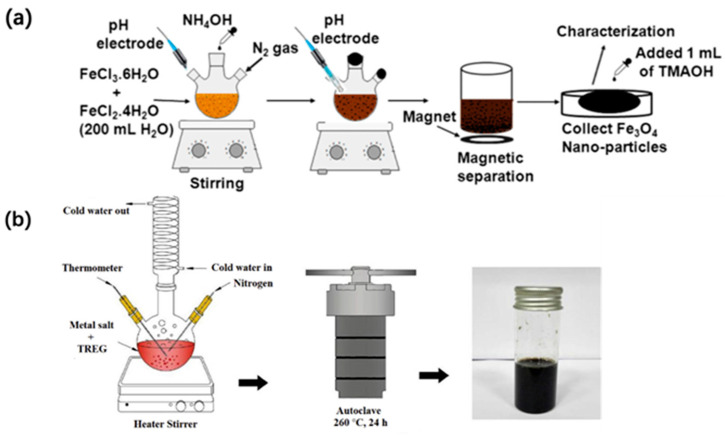
Preparation procedures of Fe_3_O_4_ by (**a**) coprecipitation and (**b**) solvothermal synthesis. (Reprinted from J. Colloid. Interface Sci. 468, Rajput, S.; Pittman Jr., C.U.; Mohan, D. Magnetic magnetite (Fe_3_O_4_) nanoparticle synthesis and applications for lead (Pb^2+^) and chromium (Cr^6+^) removal from water, 334–346. Ref. [65], Copyright © 2022 and J. Alloys Compd. 816, Fotukian, S.M.; Barati, A.; Soleymani, M.; Alizadeh, A.M. Solvothermal synthesis of CuFe_2_O_4_ and Fe_3_O_4_ nanoparticles with high heating efficiency for magnetic hyperthermia application, 152548 Ref. [153], Copyright © 2022 with permission from Elsevier).

Co-precipitation is the conventional method of synthesizing Fe_3_O_4_. First, Fe(II) and Fe(III) are mixed at a ratio of 1:2, and the resulting black powders are achieved once the solution pH becomes neutral [60]. Although this method is cheap and convenient, the final particle possesses sharp diffraction peaks in the X-ray diffraction (XRD) patterns, revealing that Fe_3_O_4_ is a type of bulk material. For Fe_3_O_4_ adsorbents, both physical adsorption and chemical reduction properties have been reported as removal mechanisms. Advanced X-ray photoelectron spectroscopy (XPS) and XRD were used to analyze the lattice structure and elemental valence state, before and after adsorption. For Cr(VI) anions, a Cr(III) oxyhydroxide or hydroxide-phase passive layer was formed on the surface of magnetite, which preferentially reacted with magnetite (111) [144]. In addition, the large surface area and abundant functional groups provided many active sorption sites. Thus, highly dispersed nanoparticles play a key role in the exposure of these sorption sites. To obtain smaller-sized particles, Rajput et al. added tetraethyl ammonium hydroxide (TEAOH) to the Fe_3_O_4_ product, to prevent agglomeration (Figure 3) [65]. Yusoff et al. [145] found that smaller magnetite particles were easier to obtain when the pH of the co-precipitation solution was greater than 12. After treatment with amino silane groups, the kinetic stability of this magnetic particle can be further developed [59]. Other approaches have also been reported for the preparation of magnetite, including the oxidation of Fe(II) by oxygen, or the reduction of Fe(III) by reducing bacteria [15,64].

The influence of the solution pH on the production of magnetic material adsorbent has been further studied. Gnanaprakash et al. [146] investigated how the initial pH of the salt solution influences the synthesis of magnetite. Additionally, the results showed that 100% spinel iron oxide was formed when the salt solution was below 5; as the pH was increased from 5.7 to 6.7, the percentage of goethite rose from 35% to 78%. The final pH is known to control the nucleation and growth of magnetite nanoparticles and can affect the size and saturation magnetization. In a certain pH range, the protonation/deprotonation of Fe–OH takes place, which can hinder the aggregation of magnetite particles, due to the combined steric and electrostatic stabilization [147,148]. Besides, the solution pH after adsorption treatment also plays a critical role in recycling performance. By adjusting the proton concentration, the surface charge of magnetite changes; thus, the pollutant can be desorbed by electrostatic forces [149].

Magnetite nanoparticles can also be directly synthesized by the thermal decomposition of Fe(III) acetylacetonate (Fe(acac)_3_) in tris(ethylene glycol), wherein the resultant average crystallite size is approximately 10.7 nm [150]. However, the final magnetite product from ferrous oxalate dihydrate, after heat treatment, is still a bulk material, even at temperatures below 90 °C [151]. Thus, the size distribution and crystallinity are mostly dependent on the Fe-containing precursors. For example, organometallic iron compounds, such as Fe(III)-acetylacetonate and Fe(III)-N-nitroso phenylhydroxylamine, are mixed in organic solvents, such as oleic acid and oleylamine. The stabilizer may inhibit the nucleation and growth process of iron oxide, so that it is well controlled in size and shape.

During the thermal decomposition, the radius of the nanoparticles can be controlled by tuning the reaction temperature and the mass ratios of the solvent reagents. Lassenberger et al. [152] further optimized monodisperse iron oxides using oleic acid as a surfactant, and the results showed that a fast heating rate accelerates the nucleation and growth of iron oxide particles. In contrast, a wide size distribution and larger particle size can also occur when the annealing duration is prolonged.

### 2.2. Attaching Pre-Synthesized Magnetic Particles and Adsorbents

**Figure 4:**
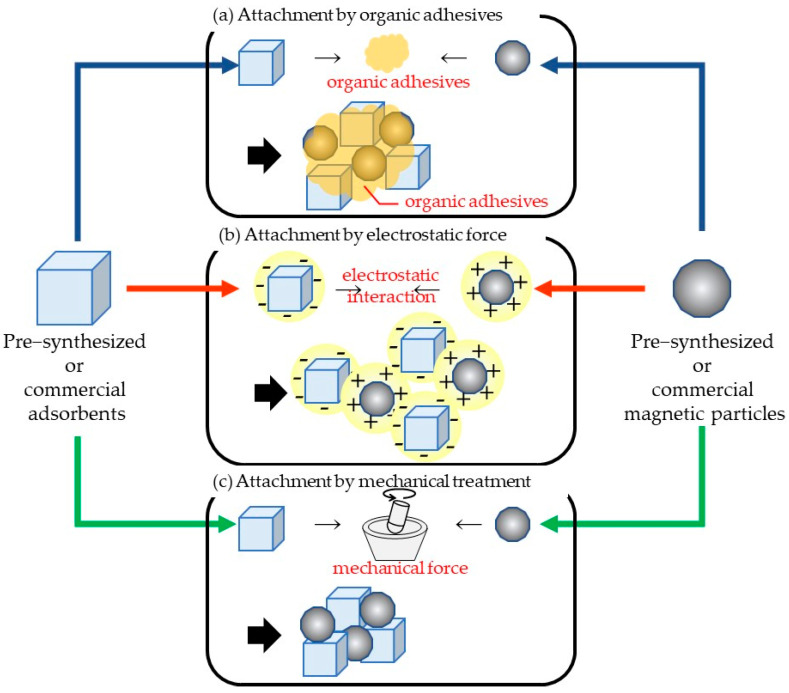
Illustration of attachment methods between adsorbents and magnetic particles.

Because the adsorption performance of magnetic materials is rather low, many researchers have sought to embed magnetic properties into well-known adsorbents. The attachment of pre-synthesized adsorbents and pre-synthesized magnetic materials is one such approach. Several methods, including attachment by organic adhesives, electrostatic interactions, self-assembly, crosslinking reactions, and mechanical bonding, can be used in this approach. Pre-synthesized materials include commercial products, apart from the ones developed in laboratories. The attachment methods are summarized in Figure 4.

#### 2.2.1. Attachment Using Organic Adhesives

One of the proposed methods is to simply attach pre-synthesized magnetic particles to pre-synthesized adsorbents. In 2006, Nah et al. [67] bonded commercial zeolites (adsorbents) with commercial Fe_3_O_4_ (magnetic particles), by adding them into a mixture of urethane and thinner, followed by vacuum drying and ball milling. The urethane functioned as an adhesive and bonded the zeolite and magnetite. The adsorption performance of Pb(II) in magnetic zeolite, tested at pH 5.0, was found to be 133 mg/g. Unfortunately, the adsorption performance was approximately half that of the original zeolite. Polymeric materials have been widely used as adhesives. Minh and Lebedeva [45] reported the attachment of alkali-treated coffee waste to Fe_3_O_4_, using polyvinyl alcohol (PVA), in 2018. Fe_3_O_4_ was dispersed in a 2% PVA solution, heated to 80 °C, and coffee waste, pretreated with sodium hydroxide (NaOH), was added, followed by filtering and drying. The obtained coffee waste/Fe_3_O_4_ composite adsorbent showed a magnetization of 21.5 emu/g and a maximum adsorption of 227 mg/g against methylene blue, at 45 °C. Although this method is very simple and easy, the adverse effect of adhesives attached onto the adsorbent surface must be considered.

#### 2.2.2. Electrostatic Interaction

Another method of attaching adsorbents to magnetic materials is electrostatic interaction. Using this method, Li et al. [37] synthesized magnetic lignin-based hollow microspheres (LHMs). The attached LHMs were prepared by self-assembly, with co-precipitated Fe_3_O_4_ through electrostatic interactions. Additionally, they stated that some of the Fe_3_O_4_ nanoparticles could also be immobilized by entering the holes of the LHMs. The adsorption capacity results showed that the magnetic LHM from larch lignin exhibited better adsorption properties for methylene blue and Rhodamine B, which were 31.23 and 17.62 mg/g, than that for poplar lignin, which were 25.95 and 15.79 mg/g, respectively. The saturation magnetization of magnetic LHM was about 22.7 emu/g, which could be easily collected by a magnet. In an experiment on the attachment between cellulose and γ-Fe_2_O_3_ nanoparticles, Luo et al. [154] reported that iron oxide nanoparticles can be strongly attached through interaction with the electron-rich oxygen atoms of hydroxyl groups. The dependence of the ionic state on pH values implies that the adhesion strength of materials that use electrostatic interactions alone may be questionable.

#### 2.2.3. Liquid Self-Assembly Method

Metin et al. [36,155] prepared graphene oxide (GO)/Fe_3_O_4_ composites using a liquid self-assembly method. The modified Hummers method and thermal decomposition of iron salt were used to prepare GO and Fe_3_O_4_, respectively. The composite, developed for Schottky diode applications, has found applications in water treatment. The composite showed a saturation magnetization of 16 emu/g. The adsorption performance was measured using methylene blue (MB) as the adsorbate and showed a maximum capacity of 172.6 mg/g. The adsorbent maintained an adsorption performance of ~80% after five cycles. Guo and Sun [156] reported that the self-assembly method applied to graphene and FePt nanoparticles seemed to stem from the graphene’s p-electron polarization, from graphene to FePt, via a possible coordination bonding, due to the close contact between the two materials. Because Metin et al. referred to their report for the self-assembly method, such polarization shall affect the attachment of the magnetic particle and adsorbent; however, further investigation is still needed to understand this mechanism.

#### 2.2.4. Crosslinking Reactions

Another method used for attaching materials is the crosslinking reaction. Hosseini et al. [157] synthesized amine-functionalized magnetic core-coated carboxylated nanochitosan shells, by coupling an amine with carboxylic groups. Chitosan nanoparticles were carboxylated with citric acid (NCS–COOH), Fe_3_O_4_ nanoparticles were functionalized with amines, using N^1^-(3-trimethoxysilylpropyl) diethylenetriamine (NH_2_–Fe_3_O_4_). Fourier transform infrared spectroscopy (FT-IR) results indicated the successful linkage of NCS–COOH on the NH_2_–Fe_3_O_4_ surface, and transmission electron microscope (TEM) images showed that a core shell structure, with NH_2_–Fe_3_O_4_ particles, encapsulated in the NCS–COOH shell was formed. The authors state that high stability over a wide range of pH and dispersity in hydrophilic solvents enables the amphoteric NH_2_–Fe_3_O_4_@NCS–COOH to be a practical candidate for various purposes, including wastewater treatment. The coupling of an amine with a carboxylic acid to form an amide bond is the most popular chemical reaction, which is also used for drug discovery [158]. This technique is effective for the attachment of adsorbent and magnetic particles.

#### 2.2.5. Mechanical Attachment

Others sought the possibility of attaching or embedding magnetic particles to adsorbents by mechanical treatments, such as ball milling. Galhoum [75] prepared magnetic nanocomposites of poly (glycidyl methacrylate) (PGMA) derivatives and nano-sized Fe_3_O_4_. Fe_3_O_4_ was prepared by co-precipitation, followed by heating at 80 °C. High-energy ball milling of the two materials was performed at 700 rpm for 60 min (including 1 min stop every 20 min). This treatment was reported to form a core–shell magnetic nanocomposite. The grafting of aminoalkylcarboxylate and aminoalkylphosphonic ligands led to an increase in sorption capacities, due to the specific reactivity of carboxylate and phosphonate. The adsorption against U(VI) for aminoalkylcarboxylate and aminoalkylphosphonic ligand-grafted magnetic PGMA, at the optimum pH of ~4.0, were 122.9 mg/g and 147.0 mg/g, respectively. A similar technique was adopted for magnetized polyethylene composites, by Mohebbi and Farajzadeh, using planetary ball milling [42]. They milled magnetic particles obtained from sand, with polyethylene powder at a ratio of 50:50 (%, *w*/*w*), and then subjected them to ball milling for 1.5 h, at a rotational speed of 900 rpm. They insisted that the heat generated by the mechanical collision melted the polyethylene, realizing a strong attachment between the two materials. The prepared composite showed a saturation magnetization of 28.43 emu/g and enabled the extraction and pre-concentration of some pesticides from fruit juices.

#### 2.2.6. Unclear Attachment Methods

Chen et al. [78] combined pre-synthesized nano Fe_3_O_4_ with pre-synthesized layered double hydroxide (LDH) nanocrystals, by simply mixing suspensions of the two materials, followed by sonication and collection by a magnet. The LDH was prepared by hydrothermal treatment, and Fe_3_O_4_ was prepared by co-precipitation. This nanohybrid magnetic adsorbent showed a high performance of 505 mg/g against Congo Red (based on LDH). The magnetic adsorbent showed a quick uptake of 96% of the capacity within 5 min and maintained a capacity of over 80% of the initial performance after four cycles. Similarly, Fungaro et al. [69,70,80] prepared a composite of zeolite, synthesized from fly ash and Fe_3_O_4_, obtained from co-precipitation. The zeolite was slowly added to a suspension of Fe_3_O_4_ particles and subsequently washed, milled, and dried. The resulting product was found to be easily attracted by a magnet; however, the adhesion force between the two materials was not clarified. Adsorption of Zn(II) [69], U(IV) [70], and two types of dyes, reactive orange 16 and indigo carmine [80], were tested. All adsorbates showed Langmuir-type adsorption, with maximum capacities of 30, 22.4, 1.06, and 0.583 mg/g, respectively. Bessa et al. [71] also synthesized magnetic zeolite by simply mixing Fe_3_O_4_, prepared by precipitation/partial oxidation, and hydrothermally synthesized zeolites A and P at a 1:3 mass ratio, macerated, and dispersed in distilled water, at 80 °C for 1 h. The saturated magnetization was ~25.5 and ~17.5 emu/g and adsorption performance against Ca(II) was 54 and 51 mg/g for zeolite A and P, respectively. Gaffer et al. [159] further extended this technique and attached chitosan as a secondary adsorbent on a magnetic adsorbent, to form magnetic zeolite–natural polymer composites. The Cr(VI) removal efficiency was 98% at pH 2, when the initial Cr(VI) concentration was 200 mg/L. Many researchers [68,69,70,71,72,73,74,75,80] utilize this technique, but the detailed adhesion mechanism needs further clarification.

### 2.3. Synthesis of Magnetic Particles on Adsorbents

Many different materials, including active carbon [102], carbon nanofiber aerogels [108], nanotubes [92,95], reduced graphene oxide [103], LDH [159], bentonite [100], and MoS_2_ [101], have been studied as potential adsorbents. However, these adsorbents cannot be easily separated after utilization, which restricts their application and development. The introduction of magnetic particles onto adsorbents could be an effective method to facilitate facile separation. Therefore, researchers have attempted to develop a series of methods to synthesize magnetic particles on pre-synthesized adsorbents. A summary of typical magnetic adsorbents with synthesis techniques and properties has been presented in Table 1.

#### 2.3.1. Co-Precipitation

This common method can be used to synthesize magnetic particles on adsorbents. During the co-precipitation process, the magnetic nanoparticles are incorporated with extensively investigated materials, such as active carbon, nanotubes, reduced graphene oxide, zeolite, LDH, and MoS_2_. Oliveira et al. [28] reported the introduction of maghemite into commercial NaY zeolite, to produce a novel adsorbent. The bulk magnetization was converted from the original value of 18 to 33 J/T·kg. Uniformly distributed small nanoparticles (3–6 nm) can also be obtained in a cubic zeolite matrix [160,161]. Despite the direct mixing of the precursors with zeolite, 1-butyl-3-methylimidazolium tetrachloroferrate ([bmim]Cl/FeCl_3_) ionic liquid has been used to immobilize magnetic NaY zeolite. However, the magnetic particles, by occupying the entrance of the zeolite porous structure, prevent the diffusion of pollutants, especially macromolecules or organic dyes [162].

Humic acid, coated on the surface of magnetite as a sorbent, is an environmentally friendly material, and the structure of the magnetite core, with a humic acid shell, has better adsorption performance in an acid condition [81]. Other surface modifiers, such as oleic acid [89], functionalized chitosan [85,86], polyacrylic acid [40], lignosulfonate [84], and activated sericite clay [98], to some extent, could enhance the dispersion of magnetic nanocomposites, because they are widely spaced. In this way, the size of the nanocomposites can be controlled on a small scale, lower than 10 nm.

Recently, more complex hybrid adsorbents have been synthesized using the co-precipitation method. Wan et al. [91] reported the results of a humic acid/L-cysteine-codecorated magnetite that has complex properties, resulting from functional groups, ion exchange, and negatively charged surfaces. In another study, Amini et al. [87] reported a novel composite, GO/Fe_3_O_4_/OPO_3_H_2_/PCN-222, that can extract U(VI) in 3 min, with a capacity of 416.7 mg/g. It took three steps to prepare the adsorbents; therefore, large-scale production was unfeasible and not cost-effective. It is worth noting that during this process, some particles were first nucleated into clusters and then aggregated together. The support materials can provide a large surface area for adhesion; however, the adhesion largely involves physical bonding, which can trigger problems, such as leaching and desorption.

#### 2.3.2. Solvothermal

The solvothermal/hydrothermal method provides a simple, direct, and low-temperature method of obtaining nanoparticles with a narrow dispersion and includes an alternative method of calcination at mild temperatures, to promote crystallization. After heating in a Teflon-lined stainless steel autoclave at 200 °C, a crystalline cubic spinel structure of Fe_3_O_4_ was successfully obtained on the surface of the mesoporous carbon in the composite [104]. In another similar study, Fe_3_O_4_/γ-Fe_2_O_3_ was loaded with active carbon, smaller in size [96]. A titanium dioxide-coated magnetic hollow mesoporous silica sphere, with a high surface area, produced by Wu et al. [163], could be used to efficiently and quickly capture phosphopeptides from peptide mixtures.

**Figure 5:**
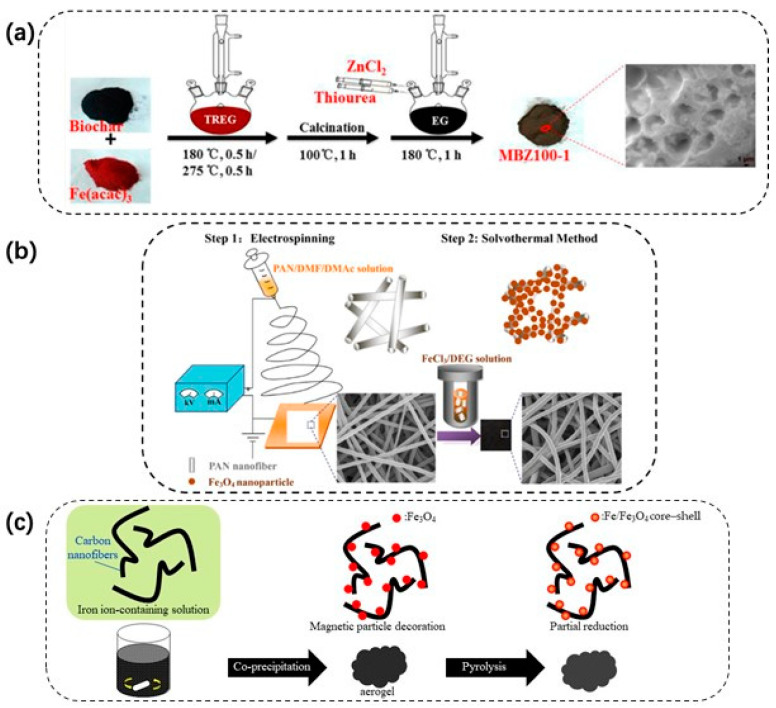
Representative preparation procedures of (**a**) magnetic biochar/ZnS, (**b**) Fe_3_O_4_/PAN composite nanofibers (NFs), and (**c**) magnetic carbon fiber aerogels. (Reprinted with permission from (**a**) ACS Sustainable Chem. Eng. 2015, 3, 1, 125–132 Ref. [164] Copyright © 2022 American Chemical Society and (**b**) ACS Appl. Mater. Interfaces 2015, 7, 14573−14583 Ref. [105] Copyright © 2022 American Chemical Society.).

One-dimensional electrospun nanofibers, with large specific surface areas and high porosities, are connected to each other, making it easy to extract them from water. Liu et al. [105] loaded cubic phase magnetite particles on polyacrylonitrile fibers, through a “two-step” process—electrospinning first and solvothermal next—without changing the morphology and structure of the spinning (Figure 5). Charpentier et al. [39] improved the chitosan-doped Fe_3_O_4_ adsorbent, using carboxymethyl chitosan. The colloidal magnetic nanoparticles were synthesized via a “one-step” versatile solvothermal method and a simultaneous removal of Pb(II), Cu(II), and Zn(II) was achieved, owing to the chain flexibility and high concentration of chelating groups from carboxymethyl chitosan. Size-controlled magnetic nanoparticles can be synthesized by a solvothermal method, using surfactants. These protective agents can prevent particles from aggregating. However, the size can range from 10 to 200 nm, and the interaction between surfactants and magnetic particles needs further research.

#### 2.3.3. Thermal Decomposition

Thermal decomposition is another facile approach for obtaining efficient adsorbents. Nanoparticles are formed in situ during the heating process and used to absorb directly, without any post-treatment. Recently, a new type of calcined magnetite-activated carbon/MgAl-LDH was prepared and characterized. It effectively removed low-concentration iodide ions (I^−^), through the “memory effect” [82]. The magnetic adsorbent and layered double oxide were prepared in the same step, making it an efficient and convenient synthesis method. Although thermal decomposition can result in a uniform size distribution and highly crystallized magnetite, it is highly energy intensive. Other essential one-pot methods should be investigated in future studies. The introduction of magnetic particles on adsorbents may occupy the active sites of adsorbents, leading to a decrease in the adsorption properties. These issues should be well addressed for practical applications.

### 2.4. Synthesis of Adsorbents on Magnetic Particles

**Figure 6:**
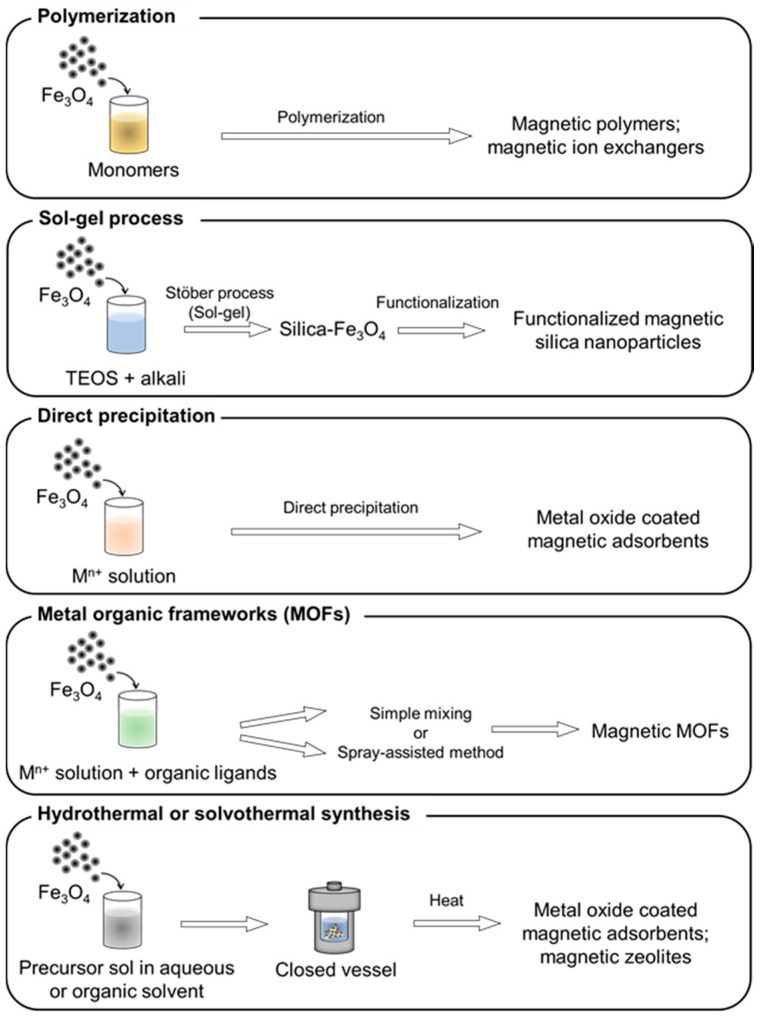
Brief procedures of conventional synthesis methods for adsorbents on magnetic particles.

Magnetic adsorbents can be prepared in a route opposite to the one described in Section 2.3, by synthesizing the adsorbent onto magnetic materials. In most cases, these are prepared by synthesizing adsorbents in the presence of pre-synthesized magnetic particles. The combination of magnetic particles with inorganic or organic adsorbents/ion exchangers was carried out in different ways, depending on the target adsorbent (Figure 6). Although Fe_3_O_4,_ pre-synthesized by conventional co-precipitation or solvothermal methods, is the most commonly used magnetic particle, some researchers have used commercialized magnetite for convenience, with an optimal particle size.

#### 2.4.1. Polymerization

Polymeric substances, applied as adsorbents or ion exchangers, can be directly grafted onto magnetic iron oxide surfaces. Polymer-coated Fe_3_O_4_ is prepared by polymerization, depending on the target polymer. A simple protocol, such as suspension of a mixture of Fe_3_O_4_ and monomers by continuous stirring, was used for grafting at room temperature, under cooling, or at an elevated temperature. For example, Bolto et al. [24] synthesized polyacrylic acid-grafted Fe_3_O_4_ as an ion exchanger in wastewater treatment; Wei et al. [134] presented a polymer-coated Fe_3_O_4_ by the self-polymerization of dopamine on Fe_3_O_4_–COOH at room temperature, for organic dye adsorption. The poly(m-phenylenediamine) layer provided predominant amino groups as adsorption sites for Cr(VI), resulting in a fivefold increase in the maximum Cr(VI) adsorption capacity, compared to that of bare Fe_3_O_4_ [113]. Another example of grafting polymers at a low temperature is the oxidative copolymerization of microbial extracellular polymeric substances (EPS) on Fe_3_O_4_, via interaction between Fe and O and C=N on EPS, by continuous stirring at 0 °C [124]. The magnetic polymer was used for the adsorption of Ag(I) and showed a slightly improved maximum adsorption capacity compared to EPS, owing to a higher surface area. Another polymer, sulfonated polystyrene, required an elevated temperature to polymerize on Fe_3_O_4_, which was then tested for Se adsorption in water samples. Sulfonated polystyrene–Fe_3_O_4_ was used as a detection tool by magnetic immobilization in a microchannel, to perform on-chip magnetic solid-phase microextraction, with subsequent instrumental analysis [165].

#### 2.4.2. Sol–Gel

The sol–gel process is widely used for coating silica shells on Fe_3_O_4_ cores, to obtain magnetic core–shell structures. The silica shell can prevent the possible loss of magnetism, due to oxidation of the magnetic oxide core, and can be modified with various functional groups. The Stöber method, a modified sol–gel process for preparing silica, is commonly used [166]. The process is initiated by the hydrolysis of tetraethyl orthosilicate (TEOS) in the presence of ethanol, water, and ammonia, with subsequent co-condensation of silicic acid, to form silica. Sodium silicate can be used for the precipitation of silica in acidic media. In the presence of the hydroxy groups on Fe_3_O_4_, a silica layer can be formed on the surface of Fe_3_O_4_ by continuous stirring, under an N_2_ atmosphere. Some additives, such as glycerol, were used as porogens, in order to increase the pore size of silica [167], contributing to the occlusion of Fe_3_O_4_ [122]. Further functionalization of silica with various silane coupling agents has been reported. Octadecyl or C18, a long-chain hydrocarbon group, was grafted onto silica–Fe_3_O_4_ through alkylation. Jiang et al. [129] used dimethyl octadecyl chlorosilane as a silane coupling agent, to functionalize an ultrafine silica–Fe_3_O_4_ simple core–shell with C18, for the adsorption of Sudan dyes in water. In another study, Li et al. [139] synthesized C18-functionalized interior pore-wall mesoporous silica, for the adsorption of phthalates in water, by mixing Fe_3_O_4_ with TEOS and n-octadecyltriethoxysilane. Here, the cationic surfactant cetyl trimethylammonium bromide (CTAB) was employed as the interior wall template. Partial silanol groups of silica were shielded from the C18-functionalized interior by ion pairing with CTAB. Finally, CTAB was removed, and the interior wall structure of C18-functionalized silica was obtained. In this manner, the adsorbents had greater dispersibility in water than the simple core–shell structure. These two types of C18-functionalized silica–Fe_3_O_4_ showed good magnetic separability, with high adsorption performance, and were utilized as adsorbents in the magnetic solid-phase extraction of organic pollutants. Amino is another widely employed functional group that provides electrostatic interactions with ionic adsorbate species. Huang et al. [112] functionalized silica–Fe_3_O_4_ with an amino group, by simply refluxing pre-synthesized silica–Fe_3_O_4_ with 3-aminopropyl trimethoxysilane (APTMS) for 8 h. The obtained product had a microspherical shape and high saturation magnetization (M_s_ = 60.6 emu/g), which was easily trapped inside a knotted tube, for the online speciation of Cr(VI,III) in water samples. Zhang et al. [117] prepared the same type of magnetic amino adsorbent and found that the amount of APTMS used affected the morphology of the product. An excess amount of APTMS was found to accelerate the hydrolysis rate of TEOS, due to its basicity, resulting in faster precipitation of silica. The faster precipitation led to an incomplete coating of silica on Fe_3_O_4_. However, well-coated spherical core–shell particles have been used for Pb(II) removal from water. Wang et al. [127] synthesized acid-resistant magnetic adsorbents for the removal of Sb(III) from strong acid solutions. The adsorbent contains an Fe_3_O_4_ core, protected by a multilayer shell, including silica, and a hydrophobic methyl group, terminated with amino methylene phosphonic acid as the adsorption site. The adsorbents showed high Sb(III) adsorption performance in strong acid media, in the presence of foreign ions. The amino groups can be further modified with various active sites for the removal of different adsorbates. For example, Huang et al. prepared amino-silica–Fe_3_O_4_ and modified it with various functional groups, such as sulfur-doped amide-linked organic polymers for Hg(II) adsorption [123], covalent organic frameworks (COFs) for diclofenac sodium adsorption [141], and recently sulfonic acid-functionalized covalent organic polymers for the removal of malachite green [136]. These functional adsorbents showed satisfactory adsorption performance and a good magnetic response, with M_s_ in the range 15−20.2 emu/g. Functionalized silica–Fe_3_O_4_ adsorbents for the removal of multi-elements have been developed. Examples include amino benzyl EDTA, immobilized on carboxylic-coated silica–Fe_3_O_4_, for chelating Cu(II), Co(II), Cd(II), and Pb(II) in water [168] and benzyl group-functionalized chitosan, modified on silica–Fe_3_O_4_, to remove organic dyes [133]. Surfactants have been used for the surface modification of silica–Fe_3_O_4_ because they possess both hydrophilic and hydrophobic moieties. Karatapanis et al. [137] utilized a cationic surfactant, cetylpyridinium bromide (CPBr), to modify the surface of silica–Fe_3_O_4_. The positive charge of CPBr attracted a negative charge on silica, in the pH range of 6 to 9, whereas the hydrophobic tails of CPBr served as the adsorption sites. The adsorbents were tested for the adsorption of six heavy metal ions in water samples, after complexation with 8-hydroxyquinoline. Sodium dodecyl sulfate (SDS), an anionic surfactant, is another example of surface modification with surfactants. Adivi et al. [140] prepared a magnetic adsorbent for the removal of phenazopyridine, a cationic drug, from water samples, by initially functionalizing silica–Fe_3_O_4_ with amino groups, with subsequent electrostatic binding to the negative heads of SDS. The hydrophobic tails of these SDS molecules interacted with the surrounding SDS tails and were finally caged in agarose. Negative charges in SDS and the hydroxy groups on agarose acted as adsorption sites for phenazopyridine. Recently, silica, apart from serving as a protection layer or functionalization bridge, has also been used as a hard template for the crystallization of titanium oxide-based nanosheets. Zhao et al. [126] synthesized amorphous TiO_2_ on silica–Fe_3_O_4_, using the Stöber method. Then, the silica layer was removed by dissolution in a strong alkali solution, followed by recrystallization of the H_2_Ti_2_O_5_ nanosheet, by redissolving TiO_2_ in diluted hydrochloric acid. After calcination at 400 °C, the crystals became flower-like nanosheets. Finally, amidoxime was grafted onto the magnetic flower-like nanosheet for the removal of U(VI) from seawater samples. The adsorbents, although obtained from a complicated process, showed good magnetic response, with an M_s_ of 15.19 emu/g.

#### 2.4.3. Direct Precipitation

The precipitation of inorganic compounds on magnetic particle surfaces was employed to prepare magnetic inorganic adsorbents. The adsorbents precipitate and attach to the surface of the magnetic particles, by simple agitation. Wang et al. [114] utilized the Stöber method to precipitate aluminum hydroxide (Al(OH)_3_) on the surface of Fe_3_O_4_, by dispersing Fe_3_O_4_ particles with aluminum isopropoxide, at room temperature. Alumina-coated Fe_3_O_4_ was obtained by calcination of the precipitate, collected at 500 °C, and subsequently trapped in a microcolumn, using an Nd–Fe–B magnet. It was then used as a solid support for SDS to further bind with 1-(2-pyridylazo)-2-naphthol as the adsorption site for Co(II), in a lab-on-valve sequential injection analysis system. Amorphous calcium silicate (CaSiO_3_) and manganese dioxide (MnO_2_)-coated Fe_3_O_4_ were prepared separately by Briso et al. [169], who evaluated it for the removal of multi-elements from acidic mine water. They found that approximately 90% of the heavy metal ions were removed by using only CaSiO_3_-coated Fe_3_O_4_ in the first step, whereas the MnO_2_-coated Fe_3_O_4_ decreased the concentration of toxic metal ions to below the permissible contamination levels, in the second step. In a more recent study, the precipitation of lanthanum hydroxide (La(OH)_3_) on the surface of Fe_3_O_4_ was accomplished on a kilogram-scale, by Ahmed et al. [109]. The preparation was performed in a tank, with continuous stirring, and using sodium hydroxide as the precipitating agent, at 50 °C. After collection, the adsorbents showed a magnetic separation rate of 98% within 3 min and were evaluated for phosphate adsorption at batch scale and pilot plant scale, using river water samples. Phosphate adsorption occurs via electrostatic interaction with protonated hydroxide groups and complexation with La. A removal efficiency of 40% was achieved at the pilot plant scale, which is approximately half that of the batch scale.

#### 2.4.4. Metal Organic Frameworks (MOFs)

MOFs are utilized as adsorbents and combined with magnetic particles to improve separation performance. A zeolitic imidazole framework (ZIF-8) was prepared on the surface of Fe_3_O_4_, pre-synthesized via a solvothermal method, in the presence of citric acid. Fe_3_O_4_ was dispersed in a mixture of zinc nitrate and imidazole at 70 °C [131] or pretreated with polystyrene sulfonate, before mixing with the ZIF-8 precursors at room temperature [119]. MOF formation is initiated by the interaction between Zn(II) and carboxylate groups in Fe_3_O_4_, followed by coordination of Zn(II) with imidazole. Morphology studies of ZIF-8 modified Fe_3_O_4_ prepared by these two synthesis processes revealed an obvious core–shell structure. Their adsorption performance was evaluated by the adsorption of methylene blue [131], Pb(II), and Cu(II) [119] in water. Another type of magnetic MOF was developed for methylene blue adsorption. Fe_3_O_4_ and TiO_2_, as photocatalysts, were incorporated into Cu_3_(1,3,5-benzenetricarboxylate)_2_ or HKUST-1, via a spray-assisted synthetic process [130]. This method is one of the fast MOF synthesis methods [170]. Fe_3_O_4_ and TiO_2_ particles were pre-mixed with the HKUST-1 mother solution, before spraying through two heated nozzles. The product was collected, washed, and dried before use. The process is fast, but the crystallinity of the product obtained by the spray-assisted process was found to be lower than that of the batch process, and the incorporation of Fe_3_O_4_ particles was quite non-uniform. The adsorbents had much lower magnetic saturation than that of bare Fe_3_O_4_, due to the thickness of HKUST-1, but were separable from solutions using a simple magnet. However, the incorporated TiO_2_ did not significantly contribute to methylene blue degradation.

#### 2.4.5. Hydrothermal and Solvothermal

Hydrothermal and solvothermal methods have been widely used for the preparation of nanoparticles, especially metal oxides. When water is used as the solvent, the process is termed hydrothermal; when other solvents are used, it is termed solvothermal. The synthesis is performed in a closed reaction vessel, above the critical temperature of solvents, in the range of 130–250 °C, and high pressure (0.3–4.0 Mpa) [8,171]. Zeolites, a group of aluminosilicate compounds that are widely used as adsorbents in environmental remediation, have been combined with magnetic particles, to improve the performance of adsorbents. The preparation of magnetic zeolites using conventional hydrothermal methods with different frameworks has been reported, including P zeolite [110], ZSM-5 [115], NaA [118], and mordenite [111]. The synthesis begins by mixing Fe_3_O_4_ with a zeolite precursor solution, before transferring it to a stainless steel autoclave, followed by crystallization at specific temperatures, for a determined amount of time. In most cases, Fe_3_O_4_ and zeolite particles are attached to each other on the surface. The ability of the synthesized magnetic zeolites to adsorb monovalent and divalent metal ions in water was evaluated. Another metal oxide, MnO_2_, was also prepared by a hydrothermal process, around the Fe_3_O_4_ core, to obtain a core–shell structure, for the removal of Cu(II) Cd(II), Zn(II), and Pb(II) [27]. The hydrothermal method was applied to synthesize magnetic graphene oxide with an LDH composite, for the first time, by Zhang et al. [116]. Before being subjected to the hydrothermal process, hydroxides of magnesium and aluminum were pre-milled, using ball milling, and were mixed with graphene oxide and Fe_3_O_4_. This process is called the mechano-hydrothermal method. During the hydrothermal process, LDHs were formed with the subsequent formation of the magnetic composite. LDHs can induce the precipitation of other metal ions. Carboxylic groups and aromatic rings in graphene oxide are attractive to metal ions and organic compounds, respectively. Therefore, the composite showed simultaneous adsorption of Pb(II) and 2,4-dichlorophenoxy acetic acid, a type of pesticide, in water. Magnetic carbon-based adsorbents were prepared using a conventional hydrothermal method. Lai et al. [125] pre-mixed sucrose solution as the carbon source with Fe_3_O_4_, before the hydrothermal treatment. The collected product was then refluxed with NaOH. The FT-IR analysis showed the partial carbonization of sucrose, to obtain carbon, whereas carboxylate and f-lactonic groups were mostly found on the surface. These functional groups were able to coordinate with U(VI); therefore, magnetic adsorbents were used to remove U(VI) from water. The solvothermal method was used to directly functionalize C18 on Fe_3_O_4_ without surface modification, using silica. Zhang et al. [138] prepared C18–Fe_3_O_4_ by dispersing Fe_3_O_4_ in ethanol and toluene, before adding octadecyltriethoxysilane. The solvothermal process was performed at 120 °C for 12 h. After collection, the C18–Fe_3_O_4_ was caged in a hydrophilic barium–alignate polymer, for magnetic solid-phase extraction of polycyclic aromatic hydrocarbons and phthalate esters from water. This is an example application of magnetic adsorbents in sample preparation, prior to instrumental analysis. MOFs can also be produced by a solvothermal process in the presence of Fe_3_O_4_. Far et al. [132] modified Fe_3_O_4_, prepared by the co-precipitation method, with zirconium-based MOFs (coded UiO-66). Fe_3_O_4_ was dispersed in N,N-dimethylformamide, in the presence of UiO-66 precursors, before the solvothermal process. The obtained particles were then modified with a polypropylene imine dendrimer by continuous stirring. The final product was found to aggregate with a non-uniform shape and size and showed soft ferromagnetism, with an M_s_ of 10.5 emu/g. The polypropylene imine dendrimer was used as an adsorption site for the evaluation of anionic dye removal in wastewater treatment plants.

## 3. Recent Advancements in Synthesis Methods of Magnetic Adsorbents

**Figure 7:**
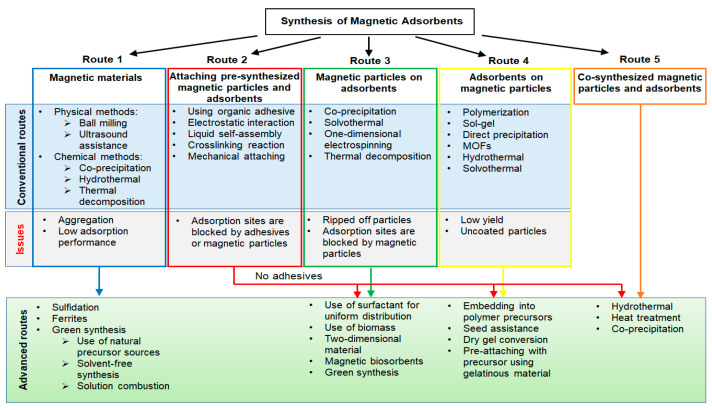
Schematic classification of the conventional synthesis methods of magnetic adsorbents covered in this review with their issues and advancements.

Although conventional methods continue to be used in a variety of studies, advanced techniques are being developed, some of which are based on conventional techniques (Figure 7). In this section, recent advancements in synthesis techniques are discussed. Advanced magnetic adsorbents in the removal of various organic and inorganic pollutants from water, according to their categorized groups, with their synthesis methods, adsorption performances, and magnetic performances are summarized in Table 2.

The improvements of these new methods make conventional synthesis less energy intensive, more efficient, and simpler, while maintaining or increasing the adsorption performance in most cases. These approaches are also important from the viewpoint of cost. The addition of magnetic properties to adsorbents increases the cost of their synthesis. However, previous reports show that the economic performance of magnetic adsorbents is already competitive in some limited cases. Oladipo et al. [172] reported that magnetic hybrids have a comparative advantage, regarding operational cost (including energy input and sludge formation), separation after spent, regeneration, and reuse in the removal of boron. Reshadi et al. [55] also claimed that rather expensive, but reusable, magnetic adsorbents have become more cost-effective than low-cost, single-use, conventional adsorbents. Therefore, magnetic adsorbents developed by the aforementioned advanced methods are expected to be competitive in wider applications.

### 3.1. Advancements of Magnetic Material Adsorbents

Recently, the development of more effective adsorbents, based on magnetic materials, has attracted much attention. In this section, we focus on the advancements in the synthesis methods for magnetic material adsorbents. A summary of the recent advancements in the synthesis of magnetic adsorbents has been shown in Table 2.

#### 3.1.1. S-nZVI

Although nZVI technology, from laboratory synthesis to environmental application, has made progress through a comprehensive amount of research, there are still two main technical obstacles to overcome, outlined here: (i) Unstable nZVI often has high surface energy and intrinsic magnetic interactions, meaning that when coupled with the van der Waals force between the particles, nZVI is easier to aggregate. The aggregated nZVI may not be as active as expected. (ii) The reducing property of nZVI may be consumed by non-target pollutants, which leads to a decrease in selectivity [198]. In the research to solve these two problems with nZVI, the sulfidation of nZVI (S-nZVI), a method of chemical modification of nZVI particles by adding sulfur compounds, recently showed that the technology is simple, inexpensive, and environmentally acceptable.

The common precipitation synthesis method of S-nZVI can be roughly divided into the following two categories: the one-step synthesis method and the two-step synthesis method. Sodium dithionite (Na_2_S_2_O_4_) is the most commonly used sulfidation reagent for one-step synthesis of n-ZVI, where iron salts are precipitated with NaBH_4_ and Na_2_S_2_O_4_, in one aqueous solution. Results have shown that core–shell structure nanoparticles are formed during the one-step method process, with a wide [S/Fe] dose range (0.07–0.4) [199]. The mixed shell structure is mainly composed of nonuniformly distributed iron hydroxides and iron sulfides (Fe_x_S_y_). Some studies have also reported the effect of sulfidation on the aggregation of nZVI and the resultant several-fold greater sequestration performance, compared with bare nZVI [142,143]. Song et al. used dithionite as a sulfur source to control the S/Fe molar ratio of S-nZVI. Their XANES results showed that the sulfidation process can effectively inhibit the formation of FeOOH on the nZVI surface and promote the crystallization of the Fe core. FeS formed on the surface can not only activate oxygen molecules into reactive oxygen species (ROS), improving the reactivity, but also suppress aggregation, due to the increased electrostatic and steric repulsion and the decreased magnetic attraction [180].

Two-step synthesis is conducted by depositing sulfur species on the surface of pre-synthesized nZVI with Na_2_S (or other sulfur-containing reagents). Unlike the one-step method that produces rough and irregular particles, the two-step method can generate nanoparticles with similar core–shell morphology and smaller sizes. Smooth spheres are usually arranged in a typical chain-like shape, with needles and plates around the core. Mangayayam et al. [200] reported that the surface layer of S-nZVI synthesized by two steps is about 5 nm thick, and the surface has defects and heterogeneous crystal orientations. In addition, post-sulfidation, using dithionite, can greatly improve the selectivity of CMC-nZVI on trichloroethylene and eliminate the reaction with water [201]. At the same time, compared to using nZVI alone, S-nZVI usually maintains a longer reaction lifespan. Dithionite dissociates to form sulfur dioxide free radicals and produces sulfite and thiosulfate, which can be used as a scavenger for electron-accepting compounds (such as O_2_) in an aqueous solution, thereby maintaining the zerovalent state of nZVI for a longer period of time [202].

#### 3.1.2. Ferrite (Mfe_2_O_4_)

In recent years, spinel ferrite has become an important magnetic nanoparticle for water treatment. The general formula of ferrite is Mfe_2_O_4_, where M could be Zn, Mg, Co, Ni, etc. Depending on the position of M(II) and Fe(III) in the crystallographic sites, there are three different types of ferrite, as follows: normal, inverse, and mixed [203]. It is also easy to separate ferrites from wastewater using an external magnetic field.

“Bottom-up” synthesis methods, including sol–gel, hydrothermal, co-precipitation, and solvothermal, have been the most frequently used methods in recent years. The adsorbent morphology, especially the capacity, is greatly affected by the different raw materials and techniques used. For example, Adel et al. [176] obtained MgFe_2_O_4_, with both microporous and mesoporous structures, by a simple sol–gel method, followed by calcination at 500 °C. Its adsorption capacity for indigo carmine dye reached 46 mg/g with spherical particles. For another magnesium–zinc ferrite composite, the increase in zinc content improved the removal efficiency of both Cr(VI) and Ni(II). The auto-combustion method enhanced the porous structure of the adsorbent [204]. In a study comparing different green synthesis methods, samples obtained by co-precipitation had better removal performance than combustion and microwave-assisted methods [63].

Fe_3_O_4_ is one of the most common ferrites and the mechanism study for the formation of Fe_3_O_4_ has been widely studied. The reaction temperature and solvent mass ratio are two factors that have a significant effect on the production of Fe_3_O_4_, not only for the adsorbent morphology but also the nanoparticle magnetism [205]. Firstly, the size of Fe_3_O_4_ particles will increase, with the increase in reaction temperatures, which has been confirmed by TEM images and calculated by the Debye–Scherrer equation, using XRD data [206]. It is believed that magnetic nanoparticles exhibit a size effect or a high surface area to volume ratio, which results in a higher metal removal adsorption capacity [207]. Secondly, the solvent used in the synthesis process is sometimes used as a multitask agent, to help produce precursors, decorate the particles or change the aggregation state of the target product. For example, in order to obtain the monodisperse nanoparticles, Xu et al. reported a simplified method of monodisperse Fe_3_O_4_, through the decomposition of iron acetylacetonate in benzyl ether and oleylamine [208]. The size of Fe_3_O_4_ can be controlled from 14 to 100 nm, by varying the heating conditions and ratios of oleylamine and oleic acid. The experiment also proved that excessive oleylamine can provide a sufficient reducing environment for the Fe precursor and promote the formation of Fe_3_O_4_ nanoparticles, at a relatively low temperature. In another study, Mohapatra et al. confirmed that the oxidation state of Fe in the prepared nanoparticles affects the superparamagnetic or nonmagnetic state, so as to influence the purity of the Fe_3_O_4_ phase [209]. Thus, both reaction temperature and solvent mass ratios are key factors for magnetite’s properties.

#### 3.1.3. Surfactant Modification

The functional modification of the surface is a common method of preventing the agglomeration of magnetic nanoparticles. Due to the steric hindrance or electrostatic repulsion, the influence from magnetic forces and van der Waals forces can be effectively suppressed. According to the surface characteristics and application scenarios, surfactant-functionalized magnetic nanocomposites can be easily classified into oil-soluble, water-soluble, and amphiphilic [210]. Chin et al. has reported a controllable method to obtain magnetite nanoparticles, using environmentally benign and non-toxic polyethylene oxide (PEO) as the solvent and surfactant simultaneously [211]. His study confirmed that the spherical-shaped Fe_3_O_4_ particles were more easily obtained when carboxylic acid (-COOH) existed during the iron acetylacetonate hydrolysis. As a common cationic surfactant, cetyltrimethylammonium bromide (CTAB) has a long, apolar chain that was used to modify palygorskite–Fe_3_O_4_. The treated palygorskite–Fe_3_O_4_ was positively charged and changed from partially hydrophobic to hydrophilic, which was favorable for the removal of anionic dyes [212]. In contrast, anionic surfactants, such as sodium dodecyl sulfate (SDS), has exhibited the hydrophobic effect and good electrostatic attraction to metal cations, such as Ni(II), Cu(II), Zn(II), and other contaminants, including norfloxacin [213,214]. Nonionic surfactants, including silica, carbon, and precious metal, can be formed as the coatings or outer shells [215,216,217]. Cendrowski et al. compared the magnetites coated with solid silica, mesoporous shell, and pristine nanoparticles. The results showed great differences in thermal and chemical stability. Due to the lack of diffusion of oxygen and hydrochloric acid through the silica structure, the thermal stability and acid resistance were both enhanced [218]. For an amphoteric surfactant, Al_2_O_3_ is an example that can react both as an acid and a base. In a study of fluoride adsorption, Chai et al. found that sulfate-doped Fe_3_O_4_ /Al_2_O_3_ exhibited a high capacity, over a wide pH range. In acidic solutions, the equilibrium pH is much higher than the initial pH; in alkaline conditions it will drop to a lower value [219]. It has shown a good amphoteric property, which is favorable to the application of adsorbents in natural water environments.

#### 3.1.4. Green Synthesis

In recent years, a number of magnetic nanoparticle synthesis methods have been developed. The particle size of nanoparticles can be controlled by great thermal decomposition of the toxic and expensive precursors and surfactant organic solvents. The high thermal energy consumption and large amount of organic waste solvents present significant environmental challenges. Thus, more solvent-less or solvent-free green synthesis methods are being investigated.

Some natural biomass has abundant functional groups and can promote the formation of magnetic particles. Coconut husks have been reported to contain phenolic substances with carboxyl groups on the surface, such as benzoic acid and caffeic acid, which could stabilize the magnetite dispersions, over a wide pH range [220]. In a similar study, iron oxide nanoparticles were mixed with tangerine peel extract. When the pH value was 4 and the adsorbent dosage was 4 g/100 mL, the maximum removal rate of Cd(II) ions was 90% [221]. The magnetic material modified by starch also has a good crystal form and maintains a good removal effect on textile dyes, other than heavy metals [178]. However, a pre-synthesized magnetic nanoparticle and aqueous solution are required to obtain the nanocomposite.

**Figure 8:**
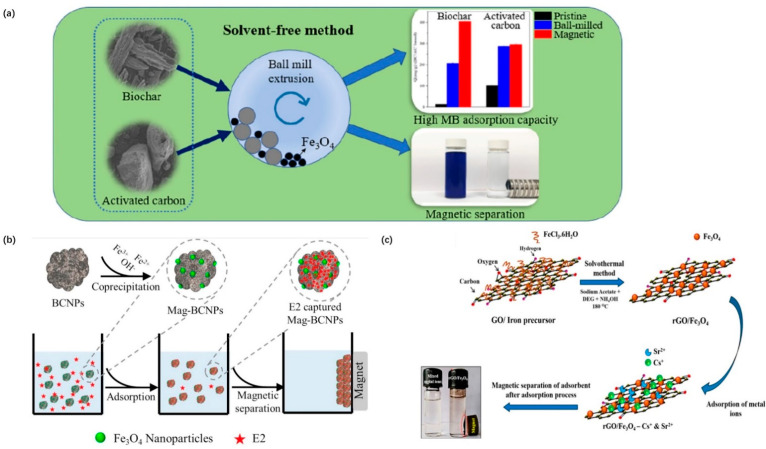
Preparation procedures of Fe_3_O_4_ loaded on (**a**) magnetic biochar and activated carbon, (**b**) nanosized biochar particles and (**c**) RGO. (Reprinted from (**a**) Sci. Total Environ. 722, Li, Y.; Zimmerman, A.R.; He F.; Chen, J.; Han, L.; Chen, H.; Hu, X.; Gao, B. Solvent-free synthesis of magnetic biochar and activated carbon through ball-mill extrusion with Fe_3_O_4_ nanoparticles for enhancing adsorption of methylene blue. 137972. Ref. [223], Copyright © 2022 and (**b**) Chem. Eng. J. 352, Dong, X.; He, L.; Hu, H.; Liu, N.; Gao, S.; Piao, Y. Removal of 17β-estradiol by using highly adsorptive magnetic bio-char nanoparticles from aqueous solution. 371–379. Ref. [185], Copyright © 2022 with permission from Elsevier and (**c**) Ind. Eng. Chem. Res. 2018, 57, 4, 1225–1232 Ref. [224] Copyright © 2022 American Chemical Society).

Using waste ferrous sulfate as the main iron source, magnetite (FeFe_2_O_4_) nanoparticles were synthesized through solvent-free reduction reactions [222]. At room temperature, the porous magnetite nanoparticles have an M_s_ of approximately 77 emu/g, which is sufficient for separation from wastewater, using an external magnetic field. Using industrial waste as a raw material to synthesize porous magnetite nanoparticles not only reduces production costs but also ensures clean production and eases environmental pressure. Sulfur dioxide produced in the reduction reaction is recycled with water to produce sulfuric acid [222]. In addition, magnetic carbonaceous adsorbents produced by ball milling biochar or activated carbon and Fe_3_O_4_ nanoparticles were obtained using a solvent-free method, and their ability to adsorb methylene blue from water was evaluated and compared [223] (Figure 8a). A possible multiple adsorption mechanism includes electrostatic interaction bonding, ion exchange, and π electronic interaction, resulting in a maximum capacity of 500.5 mg/g. Although green synthesis has many advantages, the removal efficiency and the solvent-free reaction mechanism need to be improved.

Recently, solution combustion synthesis (SCS) has become a widely adopted technique for fabricating nanomaterials, especially nano oxides, due to its attractive advantages of being simple, non-polluting, energy saving, and highly efficient. Typically, metal hydrazinecarboxylate hydrates are solid at room temperature and have a complex crystal structure. They will produce ultrafine solid oxides of corresponding metals and a large quantity of gaseous products, at relatively low temperatures (125–250 °C). Due to their exothermic and self-sustained properties, the reaction can be carried out until complete conversion, without any additional external energy input. Therefore, it is an energy-saving and sustainable synthesis method [225,226]. Xuanli Wang et al. developed a one-step SCS method to prepare Fe_3_O_4_ nanoparticles and the obtained samples have a high saturation magnetization of 89.17 emu/g and a small grain size of 57.3 nm. The flame temperature and the quantity of gaseous products released, depend on the nature of the fuel and the ratio of fuel to oxidant (φ). As the molar ratio of glycine (fuel) increases, the combustion mode changes from self-propagating combustion to smoldering combustion, and the oxide phase of SCS products sequentially changes from α-Fe_2_O_3_ → Fe_3_O_4_ → FeO [227]. The mechanisms for the formation of magnetite were further discussed by Aali et al. [228]. Glycine, urea and citric acid were used as fuels, and an overview of the results revealed that porous single-phase magnetite nano powder, with high crystallinity and saturation magnetization, was only achieved when φ  =  0.95; the highest saturation magnetization reached 99 emu/g. Magnetite and hematite were produced simultaneously in the case of urea, while in the case of glycine and citric acid, first magnetite was prepared and then oxidized to hematite. SCS also allows for the effective doping of materials and mixing with other supporting materials, to achieve large-scale production, for a wider range of applications.

### 3.2. Advancements in Synthesis of Magnetic Particles on Adsorbents

The synthesis of adsorbents with magnetic particles and high adsorption performance through conventional synthesis methods is still challenging. Therefore, an effective and green synthesis process should be developed. To maximize the efficiency of magnetic particles on adsorbents, methods that facilitate the formation of particles with uniform size, monodispersity, and non-aggregation can be applied.

#### 3.2.1. Uniform Distribution of Nanoparticles

The diffusion, transfer, fate, and environmental risks of engineered nanoparticles (ENPs) dispersed in liquid are significantly different from those of aggregation. Zhang et al. [229] showed that when the concentration of ENPs is lower than the critical micelle concentration (CMC), steric hindrance and/or electrostatic repulsion play a major role in their dispersion. When the dispersant is higher than its CMC, the dispersion of ENPs can be regarded as the “dissolution” process of the dispersant in the micelles. To overcome these agglomeration problems, associated with nanometer size, nanoparticles with high dispersion and high thermodynamic stability have been investigated.

In a recent study, sodium alginate was used to disperse nZVI to develop a new type of nZVI material [183]. Polymers chelate and coordinate ferrous ions before in situ redox, which results in an artificial “concentration” of iron into the polymer area; thus, aggregation of polymer-modified nanoparticles may be lower in an aqueous solution [230]. In addition, hydroxyl group-containing molecules that have a surface passivation effect can also inhibit subsequent crystallization, by preventing further oxidation bond formation. The increase in the number of hydroxyl groups results in a large surface energy and enhances the stability of the intermediate, consequently restraining the transformation into a crystalline form.

Functionalized, high-salt-tolerant magnetite nanoparticles were synthesized by a one-step synthesis, with sulfonated phenolic resin. Even in high-salt environments, strong anionic sulfonate groups can promote sufficient electrostatic repulsion between particles to separate them [231]. The deprotonated anionic coating can maintain the high negative charge on the surface of nanoparticles and can provide strong electrostatic repulsion, in the typical pH range of the natural aquatic environment, to maintain a highly dispersed state. However, pollutants that also have a negatively charged surface will affect adsorption and cause instability in the colloidal solution. The influence of pH, cations, anions, and humic acid on the aggregation behavior of citric acid-coated magnetite nanoparticles in the aqueous phase has been reported by Liu et al. [232].

#### 3.2.2. Bio-Derived Magnetic Nanocomposite

Many conversion schemes have been developed to use the characteristics of biomass feed to prepare adsorbents. Both biological (anaerobic digestion, hydrolysis, and fermentation) and thermal (combustion, pyrolysis, liquefaction, baking, and gasification) methods are used to convert biomass into adsorbents. Biochar from heat treatment usually has a high energy density (usually >28 kJ/g) [233]. Magnetic biochar is mainly prepared by the following three methods: calcination, co-precipitation, and pyrolysis [234]. Generally, the typical pyrolysis process can be subdivided into fast pyrolysis, slow pyrolysis, and flash pyrolysis. Co-precipitation is a bottom-up ion precipitation reaction. Calcination is a heat treatment process in which small molecules, such as H_2_O, CO_2_, and SO_2_, are removed [234]. The factors affecting the adsorption of pollutants by the magnetic biochar are summarized below.

There have been attempts to develop a magnetic biochar adsorbent to ensure a better and more effective separation of biochar particles, after the wastewater treatment process [181,233]. Further, several studies have been conducted, wherein biochar is magnetized, characterized, and applied to wastewater treatment [182,184,185,235] (Figure 8). Park et al. [236] used sesame straw raw biochar to adsorb multiple metal ions (Pb, Cr, Cd, Cu, and Zn) from wastewater. The results showed that its adsorption behavior for multi-metals was different from single-metal adsorption; in multi-metals, the adsorption of Cd was particularly impeded due to competition. However, to ensure the applicability of magnetic biochar for wastewater treatment, it is necessary to use physical and chemical conditions to simulate polluted water or use actual polluted water.

The high surface area produces rapid adsorption kinetics and, thus, a relatively short contact time. Furthermore, these nanocomposites are magnetic and can be separated from aqueous streams using an external magnetic field. Overall, magnetic nanocomposites have become a revolutionary tool for wastewater treatment, due to their unique properties and the overlap with current technologies.

#### 3.2.3. Matrice-Confined NPs

The physical confinement of nanoparticles within the structure is mainly based on weak interactions, generated by hydrogen bonds, π–π bonds, or covalent grafting, which can be achieved by in situ chemistry or post-processing methods. A physical confinement strategy is attractive as the specific reactivity of the nanoparticles is retained, and the performance can be easily adjusted by changing the size and shape of the pores, to promote the regional growth of the nanocrystals. Among them, porous carbon, mesoporous silicon oxide, aluminum oxide, and montmorillonite have been widely studied.

Silica has many advantages, such as low cost, easy preparation, good liquid dispersion, easy modification, pH resistance, and thermal stability, making it highly suitable for use in magnetic composites. Generally, the magnetic particles may be arranged on different areas of the mesoporous silicon oxides, for example, as a core, scattered distribution, or fixed/grafted on the surface or in the siliceous framework, by functionalization in the mesopores. For instance, Fe_3_O_4_ nanoparticles coated with silica and naphthoyl chloride (Fe_3_O_4_@SiO_2_@Nap) [237], porphyrin copolymer (Fe_3_O_4_ @SiO_2_-TCPP) [238], lignin (Fe_3_O_4_@SiO_2_-NH-MFL) [120], and porous aromatic frameworks (Fe_3_O_4_@SiO_2_@PAF-6) [239] have been applied to water samples, to achieve ultrafast absorption. Improved Stöber and template removal methods are used to prepare magnetic mesoporous silica nanoparticles (MSNs), which contain a Fe_3_O_4_ core and a mesoporous silica shell [240].

Compared with one-dimensional materials with lower adsorption capacity, advanced two-dimensional materials have a higher surface area and better adsorption potential. Graphene is a typical two-dimensional material. As a carbon derivative, graphene exhibits high porosity. Graphene-based adsorbents have been widely studied for water treatment applications. In recent studies, magnetic iron sulfide (Fe_3_S_4_) was synthesized via a solvothermal process. The synthesized samples were analyzed, using various characterization techniques, to understand the adsorption mechanism of Pb(II) [49]. In another study, the authors compared the removal effect of a magnetic composite consisting of one-dimensional nanofibers and two-dimensional graphene, for Pb(II) and Cr(VI), respectively. The adsorption capacities of graphene and nanofibers for Pb(II) ions were 131.40 and 42.90 mg/g, respectively. In the case of Cr(VI) ions, the adsorption capacities were 68.85 and 51.07 mg/g, respectively [241]. In addition, two-dimensional, ultrathin nanosheet-like LDHs were modified with magnetic particles [242,243]. In this work, the improved adsorption performance was due to the enhanced exposure of active sites to pollutants.

Magnetic materials based on biosorbents are considered to be highly efficient and environmentally friendly. These synergistic biomaterials can be used to modify magnetic nanoparticles with various adsorption mechanisms, for use in simple applications and to achieve reusability [244]. For instance, cholesterol improves the stability of magnetic phosphatidylcholine, by increasing the accumulation of phospholipid molecules, which may improve the reusability of the adsorbent (>8 times) for the extraction of Organochlorine pesticides (OCPs). Cholesterol-functionalized magnetic nanoparticles can be obtained using APTES and cholesteryl chloroformate modification [245]. Verma et al. [246] synthesized magnetic biosorbents from citrus (peel and pulp) biomass waste, for wastewater treatment. Samples were synthesized at 500 °C, and both As(III) and As(V) from groundwater were removed. Many new adsorbents are still in the laboratory stage. They have demonstrated the ability to adsorb pollutants from water, under different pH values, different ionic strengths, and mixed with a large amount of organic matter. The adsorption capacity and removal mechanisms are gradually being understood. In future studies, the main challenges faced by wastewater treatment may result in the inclination to investigate environmental application risk, life cycle, and long-term evolution mechanisms.

### 3.3. Advancements in Synthesis of Adsorbents on Magnetic Particles

**Figure 9:**
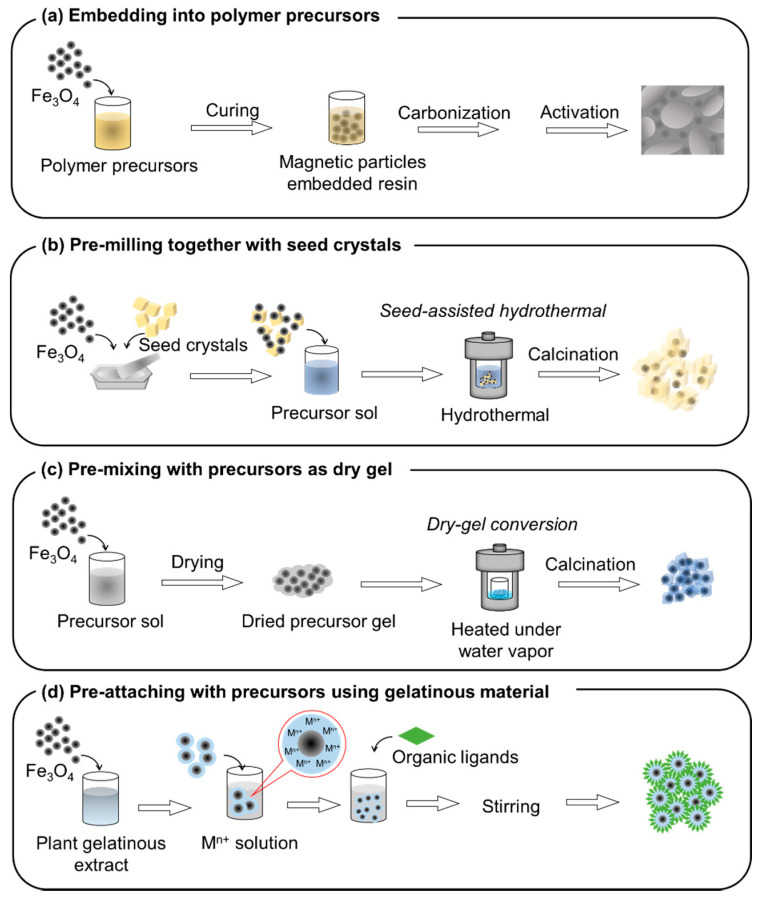
Brief procedures of the advanced synthesis of adsorbents on magnetic particles. (**a**) Embedding Fe_3_O_4_ into polymer precursors to yield magnetic activated carbon. (**b**) Pre-milling of Fe_3_O_4_ with zeolite seed crystals together to produce magnetic zeolites. (**c**) Pre-mixing Fe_3_O_4_ with zeolite precursors to form dried precursor gel before dry gel conversion to magnetic zeolite. (**d**) Pre-attaching gelatinous material coated Fe_3_O_4_ with metal ion precursor with subsequent formation of MOFs.

Efforts to synthesize well-incorporated magnetic adsorbents with uniform size and good adsorption performance are still challenging. The incorporation of magnetic particles into the adsorbents can be achieved by limiting the adsorbent formation in the vicinity of the magnetic particles. The advanced synthesis of adsorbents on magnetic particles can be accomplished by controlling or directing the growth of the adsorbent on magnetic particles, or by using pre-attached magnetic particles with adsorbent precursors. The synthesis procedures introduced in research articles, representing recent advancements in this category, are briefly depicted in Figure 9.

#### 3.3.1. Embedding into Polymer Precursors

The rigid nature of natural carbon sources limits their ability to combine with magnetic particles. Nijpanich et al. [34] produced the first study to successfully incorporate magnetic particles into small-sized activated carbon, by embedding Fe_3_O_4_ particles into epoxy resin as a carbon source. The starting materials of the epoxy resin were mixed with Fe_3_O_4_ particles, before curing at room temperature (Figure 9a). This process allowed the incorporation of magnetic particles into the carbon source, prior to the carbonization and activation process. The as-prepared magnetic-activated carbon showed no significant difference in methylene blue adsorption performance, compared to non-magnetic-activated carbon prepared by the same route. The performance, however, was higher than that of magnetic-activated carbon derived from other carbon sources. There was no negative effect due to the embedded magnetic particles on the porosity and adsorption performance of the activated carbon. Although the Fe phase was found in the final product by XRD analysis due to the reduction of Fe_3_O_4_ under the activation conditions, the adsorbents were more easily collected using an external magnet, owing to a higher magnetic susceptibility.

#### 3.3.2. Application of Seed Crystals

Recent studies have shown advancements in the synthesis of magnetic zeolites. Hagio et al. applied seed-assisted hydrothermal synthesis, an alternative but selective technique for preparing zeolites, to synthesize magnetic FAU-type [29] and MOR-type zeolites [30] for the first time (Figure 9b). In principle, with hydrothermal treatment, the addition of the target zeolite crystals as the seed into precursors can induce and accelerate the crystallization of zeolites. Therefore, a high product yield was obtained. Seed crystals act as nucleation centers for crystal growth. According to this principle, if the seed crystals are well mixed with magnetic particles, the target zeolites crystallize near the magnetic particles. Hagio et al. discovered that pre-milling seed crystals and Fe_3_O_4_ particles together, before adding to the precursors, allowed for good pre-attachment among them [30]. The growth of zeolite crystals occurred in the tiny gaps between the seed and Fe_3_O_4_ particles. Consequently, the Fe_3_O_4_ particles were completely incorporated inside the zeolite crystals. Furthermore, the amount of magnetic zeolite produced by the seed-assisted technique was found to be almost two times higher than that prepared without seed crystals. Both magnetic FAU-type and MOR-type zeolites showed good separability from solutions, using an external magnet. The magnetic FAU-type zeolite showed similar performance of methylene blue adsorption to the non-magnetic one, which revealed no adverse effects from the incorporated Fe_3_O_4_, inside the FAU frameworks.

#### 3.3.3. Pre-Mixing into Precursor Gel

Another advancement of magnetic zeolite synthesis is the utilization of the dry-gel conversion (DGC) method, first presented by Phouthavong et al. [31]. Some researchers have already introduced this technique, which crystallizes a precursor gel under heated vapor to synthesize well-crystallized non-magnetic zeolites [247,248]. In this route, Fe_3_O_4_ particles were homogenously pre-mixed into dry gel precursors before placing under heat water vapor (Figure 9c). Because the Fe_3_O_4_ particles were closely attached to the precursors in the dry gel, they were subsequently incorporated into the BEA-type zeolite during crystal growth. After the DGC, the obtained particles were collected without washing and subsequently calcinated to remove the organic template. Low waste generation is another advantage of DGC. The obtained magnetic BEA-type zeolite had a homogenous and uniform shape, size, and magnetic response. Their adsorption ability to remove methylene blue from water was similar to that of non-magnetic BEA. This indicates that the incorporated Fe_3_O_4_ particles did not clog the pores of the zeolite.

#### 3.3.4. Pre-Attaching with Precursor Using Gelatinous Material

Recently, basil seed mucilage, a plant-based product, was also used in magnetic composite synthesis technology. Mahmoodi and Javanbakht [190] prepared magnetic MOF nano-biocomposites by depositing gelatinous parts, extracted from basil seed mucilage, on Fe_3_O_4_ particles and further modifying them with ZIF-8 MOFs. To synthesize ZIF-8, a Zn(II)-methanol solution was stirred in the presence of mucilage-coated Fe_3_O_4_ particles. The Zn(II) ions were absorbed into the mucilage via electrostatic interactions (Figure 9d). Therefore, the Zn(II)-loaded, mucilage-coated Fe_3_O_4_ particles acted as nucleation centers for the growth of ZIF-8, after the addition of the organic precursor, 2-methylimidazole.

### 3.4. Co-Synthesis of Magnetic Particles and Adsorbents

**Figure 10:**
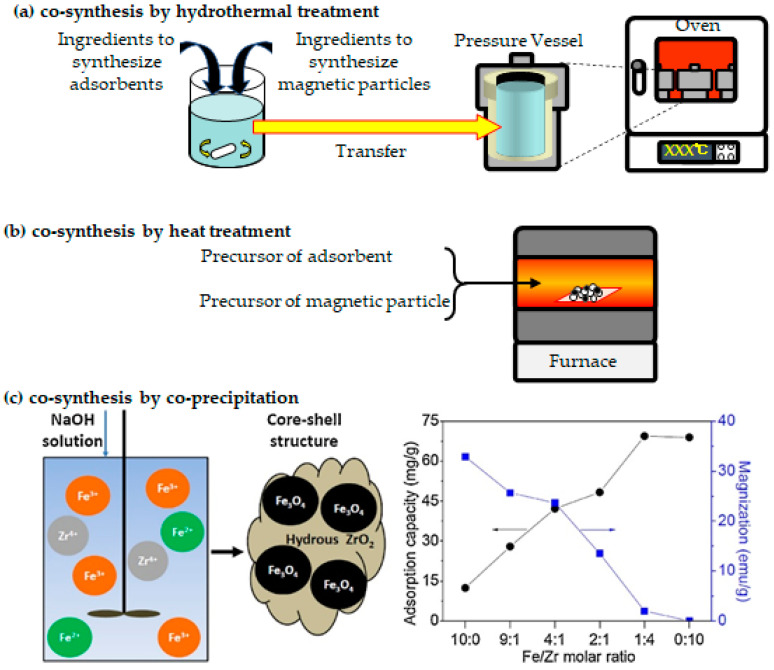
Illustrations of procedures for co-synthesis methods. (**a**) Hydrothermal, (**b**) heat treatment, and (**c**) co-synthesis methods. ((**c**) Reprinted from Appl. Surf. Sci. 366, Wang, Z.; Xing, M.; Fang, W.; Wu, D. One-step synthesis of magnetite core/zirconia shell nanocomposite for high efficiency removal of phosphate from water, 67–77. Ref. [26], Copyright © 2022 with permission from Elsevier).

In some cases, adsorbents and magnetic materials can be synthesized using a similar process. Methods to simultaneously synthesize adsorbents and magnetic materials have recently been proposed. To the best of our knowledge, this one-pot synthesis method was first introduced in the past decade. The co-synthesis methods are briefly introduced in Figure 10.

#### 3.4.1. Co-Synthesis via Hydrothermal Synthesis

Because Fe_3_O_4_ (magnetic particles) can be synthesized from alkaline solutions containing Fe(II) and Fe(III), adsorbents that crystallize from alkaline solutions have been considered for the co-synthesis of adsorbents and magnetic particles. In 2013, Aono et al. [249] prepared a composite material, consisting of Na-P1-type zeolite, and nanosized magnetite, by alkali processing from a mixed solution of fly ash and FeCl_2_ and FeCl_3_, followed by hydrothermal synthesis at 100 °C for 24 h. From TEM observations, Fe_3_O_4_ nanoparticles were observed at the grain boundaries between the polycrystalline zeolites. This was expected, as the formation of zeolite crystals is slower than the formation of nanosized Fe_3_O_4_. The resulting magnetic zeolite could achieve a 61% decontamination of soil containing radioactive Cs. Chen et al. [195] prepared magnetic MnFe_2_O_4_-sludge biochar via a one-pot hydrothermal synthesis for Pb(II) removal. Dried sludge obtained from sewage was added to deionized water and mixed with MnCl_2_·4H_2_O and FeCl_3_·6H_2_O. NaOH (5 M) was added dropwise, until the pH reached 10–10.5. This was followed by hydrothermal synthesis at 180 °C for 10 h. The washed and dried product was tested with a Pb(II)-containing solution and showed a maximum adsorption amount of 174.216 mg/g.

#### 3.4.2. Co-Synthesis via Heat Treatment

Another approach is to embed the iron ions into organic materials, followed by heat treatment in a vacuum or inert gas atmosphere. In this method, the polymeric material turns into active carbon adsorbents, and iron ions crystallize into Fe_3_O_4_ particles. Fan et al. [194] used rice husks, pretreated with a 2 M NaOH solution, and added them to ethanol, containing FeCl_3_·6H_2_O. After drying, heat treatment at 800 °C was conducted for 2 h, under a N_2_ atmosphere. Fe_3_O_4_ particles were deposited on carbonaceous material. Adsorption capacities against Cr(VI) of this magnetic carbonaceous material in 50 and 100 mg/L Cr(VI) solutions were 49.913 and 99.158 mg/g, respectively. The saturated magnetization was 77.8 emu/g. Jung et al. [33] prepared magnetic biochar/Fe_3_O_4_ nanocomposites, using brown marine macroalgae as the precursor for biochar. An electrochemical technique was used to supply the iron ions, and heat treatment was carried out at 600 °C for 1 h. The magnetic biochar possessed superparamagnetic characteristics, with a saturation magnetization of approximately 26.79 emu/g, and showed a maximum adsorption capacity of 382.01 mg/g for acid orange 7, a type of dye, at 30 °C. Although the process is one-pot synthesis, these methods require some pretreatments. Elessawy et al. [196] synthesized functionalized magnetic fullerene nanocomposites in a one-pot process, using cut PET bottle waste and ferrocene. The two materials were introduced into a stainless steel autoclave reactor, which was heated to 800 °C for 20 h, resulting in a black product. This was a facile, one-step, green synthesis route, using catalytic thermal decomposition of PET bottle waste as a precursor and ferrocene as a catalyst and precursor for magnetic nanoparticles in the composite. The nanocomposite showed saturated magnetization of 7.002 emu/g and a maximum adsorption of 833.3 and 806.5 mg/g against methylene blue and acid blue 25, respectively, at 25 °C.

#### 3.4.3. Co-Synthesis via Co-Precipitation

Co-precipitation is also extended to realize the one-pot synthesis of magnetic adsorbents. Wang et al. [26] demonstrated the one-step synthesis of Fe_3_O_4_ core/zirconia shell nanocomposites, using the co-precipitation method. Particular amounts of FeSO_4_·7H_2_O, FeCl_3_·6H_2_O, and ZrOCl_2_·8H_2_O were dissolved in doubly distilled water. Precipitation was caused by the dropwise addition of a 6 M NaOH solution, under stirring at 400 rpm, until the pH reached 7.6, and was aged without stirring at 60 °C for 18 h. The washed, collected, and dried product consisted of ball-like or hexagonal particles of Fe_3_O_4_ and cloud-like zirconia. The adsorption performance was evaluated through phosphate removal, which exhibited maximum adsorption capacities of 27.93–69.44 mg/g, when the Fe/Zr molar ratio was varied between 9:1 to 1:4, as shown in Figure 10c.

Zhao et al. [191] synthesized a magnetic Mg–Al–La composite using co-precipitation, followed by calcination for fluoride removal. Similar to the aforementioned study, a solution containing FeSO_4_·7H_2_O and FeCl_3_·6H_2_O was mixed with another solution containing Mg(NO_3_)_2_·6H_2_O, Al(NO_3_)_3_·9H_2_O, and La(NO_3_)_3_·6H_2_O, and then a NaOH solution was added dropwise to carry out co-precipitation. The Mg–Al–La hydroxide material exhibited a hydrotalcite-like structure, and iron was converted to Fe_3_O_4_ by calcination. The calcined magnetic Mg–Al–La composite showed a maximum adsorption capacity of 65.75 mg/g against fluoride.

Although the co-synthesis of adsorbents and magnetic particles is rather simple, it should be noted that it does not solve all the difficulties in the preparation of magnetic adsorbents, such as the prevention of adsorption sites by magnetic particles. Control of the structure during the synthesis and the combination of appropriate adsorbents and magnetic materials may overcome these issues.

## 4. Summary and Future View

In conclusion, the advancement in the synthesis methods of magnetic adsorbents, for the removal of substances from water streams, has been comprehensively summarized and discussed. Although numerous techniques have been developed for the preparation of magnetic adsorbents with effective adsorption performance, reviews that focus on the synthesis methods of magnetic adsorbents for wastewater treatment and the obtained material structures have not been reported, which are important for the future development of such materials. Generally, the typical synthesis methods are categorized into the following five groups: direct use of magnetic particles, attachment of pre-prepared adsorbents and pre-prepared magnetic particles, synthesis of magnetic particles on pre-prepared adsorbents, synthesis of adsorbents on pre-prepared magnetic particles, and co-synthesis of adsorbents and magnetic particles. The improvements in these new methods make conventional synthesis more energy saving, more efficient, and simpler in process while maintaining or increasing the adsorption performance. Advanced methods have overcome the negative aspects of compositing, such as the coverage of adsorption sites by the magnetic substance, through control of material structures and/or enhancing the adsorption ability of magnetic particles.

Despite the long history of magnetic adsorbents, there are still issues with their synthesis methods that need to be addressed. Primarily, the improvement of the magnetic adsorbent synthesis method to realize effective and uniform compositing is still necessary. The yields of the magnetic adsorbents are not always stated, which has a significant impact on the process simplicity and material cost. In addition, a thorough investigation is needed to enable the design and control of magnetic adsorbent structures to achieve the intensive and selective removal of specific pollutants. Moreover, the development of simulations and observations to understand the synthesis process and adsorption process is still challenging; however, it is expected that this will provide greater insight into the design of ideal magnetic adsorbents, through information on the relationship between the composite structure and performance. Finally, further improvement in reusability is essential for realizing an efficient, economical, and environmentally friendly synthesis method for magnetic adsorbents, which is required to expand applicable cases in social implementation.

## Figures and Tables

**Table 1:** Magnetic adsorbents, their conventional synthesis routes, and performance on adsorption of organic and inorganic pollutants in water.

Adsorbents	Synthesis Method	Magnetic Properties	Pollutant(s)	Adsorption or Removal Performance	Reference
**Magnetic materials as adsorbents**					
Microsized Fe	Commercial	M_s_ = 1725 kA/m	Phosphate	q_m_ = 18.83 mg/g	[59]
Nanosized Fe_3_O_4_	Sol–gel precipitation and re-crystallization	M_s_ = 477 kA/m	Phosphate	q_m_ = 27.15 mg/g	[59]
Fe_3_O_4_	Ferrite process	Not reported	Phosphate	q_m_ = 1.9–3.7 mg/g	[15]
Fe_3_O_4_	Co-precipitation	Not reported	Phosphate	q_m_ = 15.2 mg/g	[60]
Mixed Fe_3_O_4_ and γ-Fe_2_O_3_	Microemulsion	Not reported	Phosphate	Removal efficiency >95%	[18]
Mixed α-Fe_2_O_3_ and γ-Fe_2_O_3_	Dispersion-precipitation	M_s_ = 20 emu/g	Arsenite	q_m_ = 46.5 mg/g	[61]
Fe_3_O_4_	Simple mixing and sintering	M_s_ = 57.4 emu/g	As(V)	q_m_ = 20.24 mg/g	[48]
Fe_3_O_4_	Co-precipitation	M_s_ = 56.86 emu/g	As(V)	q_m_ = 44.99 mg/g	[62]
Mixed Fe_3_O_4_ and γ-Fe_2_O_3_	Co-precipitation	M_s_ = 67 emu/g	Pb(II) Cr(III) Cd(II)	q_m_ = 617.3 mg/g q_m_ = 277.0 mg/g q_m_ = 223.7 mg/g	[63]
Biogenic Fe_3_O_4_	Fe3+-reducing bacterial enrichment culture	Not reported	Co(II) Ni(II) Mn(II) Zn(II)	q_m_ = 27.44 mg/g q_m_ = 25.22 mg/g q_m_ = 26.55 mg/g q_m_ = 77.27 mg/g	[64]
Fe_3_O_4_	Co-precipitation	M_s_ = 65.33 emu/g	Pb(II) Cr(VI)	q_m_ = 53.11 mg/g q_m_ = 34.87 mg/g	[65]
**Attaching of pre–synthesized magnetic particles with adsorbents**					
Clinoptilolite–Fe_3_O_4_	Mechanical method	Not reported	Cd(II) Cu(II) Pb(II)	Removal efficiency =50% Removal efficiency =70% Removal efficiency =90%	[66]
Zeolite–Fe_3_O_4_	Using organic adhesive	Not reported	Pb(II)	q_m_ = 133 mg/g	[67]
Faujasite zeolite-CoFe_2_O_4_	Ultrasonication	M_s_ = 18.93 emu/g	Pb(II)	Removal efficiency = 99%	[68]
Wheat stalk-derived chars–Fe_3_O_4_	Simple mixing	M_s_ = 28.6 emu/g	Pb(II)	q_m_ = 179.85 mg/g	[35]
Rice husk-derived chars–Fe_3_O_4_	Simple mixing	M_s_ = 26.1 emu/g	Pb(II)	q_m_ = 95.44 mg/g	[35]
Activated carbon–Fe_3_O_4_	Simple mixing	M_s_ = 27.2 emu/g	Pb(II)	q_m_ = 43.38 mg/g	[35]
Zeolite–Fe_3_O_4_	Simple dispersion	Not reported	Zn(II)	q_m_ = 30 mg/g	[69]
Na-P1 and hydroxysodalite–Fe_3_O_4_	Mechanical method	Not reported	U(VI)	q_m_ = 22.4 mg/g	[70]
Kaolin-based zeolite A– Fe_3_O_4_	Maceration and dispersion	Ms»37.1 emu/g	Ca(II)	q_m_ = 54 mg/g	[71]
Kaolin-based zeolite P– Fe_3_O_4_	Maceration and dispersion	Ms»37.1 emu/g	Ca(II)	q_m_ = 51 mg/g	[71]
Zeolite–chitosan–Fe_3_O_4_	Simple mixing	Not reported	Cr(VI)	Removal efficiency =98%	[72]
Thiol and amine functionalized cellulose–Fe_3_O_4_	Stirring	Not reported	Pt(IV)	q_m_ = 40.48 mg/g	[73]
Hydroxyapatite/chitosan cross-linked with green tea derived polyphenol–Fe_3_O_4_	Stirring	M_s_ = 53.6 emu/g	Ni(II)	q_m_ = 112.36 mg/g	[74]
Iminodiacetate functionalized PGMA–Fe_3_O_4_	High-energy ball milling	M_s_ = 22.56 emu/g	Uranyl	q_m_ = 122.9 mg/g	[75]
Iminodiphosphonate functionalized PGMA–Fe_3_O_4_	High-energy ball milling	M_s_ = 21.14 emu/g	Uranyl	q_m_ = 147.0 mg/g	[75]
Phenol modified ZIF-8 functionalized carboxymethyl cellulose–Fe_3_O_4_	Ultrasonication	Not reported	Rb(I)	q_m_ = 109 mg/g	[76]
[Epichlorohydrin-co-triethylenetetramine]_n_-graft-CSSNa–Fe_3_O_4_ microspheres	Ultrasonication	M_s_ = 50.51 emu/g	Pb(II) Cd(II) Cu(II) Zn(II)	q_m_ = 293.38 mg/g q_m_ = 256.69 mg/g q_m_ = 277.93 mg/g q_m_ = 225.07 mg/g	[41]
NiAl LDH–guar gum polymer–Fe_3_O_4_	Ultrasonication	Not reported	Cr(VI)	q_m_ = 101 mg/g	[77]
MgAl LDH–Fe_3_O_4_	Ultrasonication	Not reported	Congo red	q_m_ = 505 mg/g	[78]
Graphene oxide–Fe_3_O_4_	Liquid-self assembly	M_s_ = 18.2 emu/g	Methylene blue	q_m_ = 172.6 mg/g	[36]
Activated carbon–Fe_3_O_4_	Ball milling	M_s_ = 33.8 emu/g	Methylene blue	q_m_ = 500.5 mg/g	[36]
Larch wood derived lignin hollow microsphares–Fe_3_O_4_	Mechanical mixing	M_s_ = 22.7 emu/g	Methylene blue Rhodamine B	q_m_ = 31.23 mg/g q_m_ = 17.62 mg/g	[37]
Poplar wood derived lignin hollow microsphares–Fe_3_O_4_	Mechanical mixing	M_s_ = 22.7 emu/g	Methylene blue Rhodamine B	q_m_ = 25.95 mg/g q_m_ = 15.79 mg/g	[37]
Silica aerogel–Fe_3_O_4_	Simple stirring	Not reported	Rhodamine B and oil	Removal efficiency =98.5%	[79]
Coffee waste–Fe_3_O_4_	Dispersion	M_s_ = 21.5 emu/g	Methylene blue	q_m_ ≈ 128 mg/g	[45]
Zeolite–Fe_3_O_4_	Simple mixing	Not reported	Reactive orange 16 Indigo carmine	q_m_ = 1.1 mg/g q_m_ = 0.58 mg/g	[80]
Polyethylene–Fe_3_O_4_	Ball milling	M_s_ = 28.43 emu/g	Pesticides	Recovery = 88–99%	[42]
**Synthesis of magnetic particles on adsorbents**					
Humic acid–Fe_3_O_4_	Co-precipitation	Not reported	Phosphate	q_m_ = 28.9 mg/g	[81]
Activated carbon/MgAl-LDH–Fe_3_O_4_	Thermal decomposition	M_s_ = 20.12 emu/g	I^−^	Adsorption efficiency = 86%	[82]
Calcined orange peel–Fe_3_O_4_	Co-precipitation and calcination	M_s_ = 14.6 emu/g	As(III)	q_m_ = 10.3 mg/g	[46]
NaY zeolite–γ Fe_2_O_3_	Co-precipitation	M_s_ = 18 emu/g	Cr(III) Cu(II) Zn(II)	q_m_ = 49 mg/g q_m_ = 87 mg/g q_m_ = 114 mg/g	[28]
Polyacrylic acid– Fe_3_O_4_/γ-Fe_2_O_3_	Co-precipitation	M_s_ = 50 emu/g	Mn(II) Co(II) Cu(II) Zn(II) Pb(II)	q_m_ = 7.97 mg/g q_m_ = 12.0 mg/g q_m_ = 19.2 mg/g q_m_ = 18.4 mg/g q_m_ = 29.8 mg/g	[40]
MoS_2_–Fe_3_O_4_	Co-precipitation	M_s_ = 35.6 emu/g	Cr(VI) Cr(III)	q_m_ = 218.27 mg/g q_m_ = 119.38 mg/g	[83]
Lignosulfonate–Fe_3_O_4_	Co-precipitation	M_s_ = 43.98 emu/g	Cr(VI)	q_m_ = 57.14 mg/g	[84]
Guanidinylated chitosan nanobiocomposite–Fe_3_O_4_	Co-precipitation	M_s_ = 43.66 emu/g	Pb(II) Cu(II) Cr(VI)	Removal efficiency =98.64% Removal efficiency =100% Removal efficiency =33.76%	[85]
Chitosan–Fe_3_O_4_	Solvothermal	M_s_ = 13 emu/g	Pb(II) Cu(II) Zn(II)	q_m_ = 243 mg/g q_m_ = 232 mg/g q_m_ = 131 mg/g	[39]
Carboxymethyl chitosan–Fe_3_O_4_	Solvothermal	M_s_ = 15 emu/g	Pb(II) Cu(II) Zn(II)	q_m_ = 141 mg/g q_m_ = 123 mg/g q_m_ = 88 mg/g	[39]
DTPA functionalized chitosan–Fe_3_O_4_	Co-precipitation	M_s_ = 35.9 emu/g	U(VI)	q_m_ ≈ 160 mg/g	[86]
Graphene oxide modified with OPO3H2/mesoporous Zr-MOF–Fe_3_O_4_	Co-precipitation	M_s_ = 8 emu/g	U(VI)	q_m_ = 416.7 mg/g	[87]
Cd^2+^ imprinted polymer on carbon nanotube–Fe_3_O_4_	Solvothermal	Not reported	Cd(II)	q_m_ = 81 mg/g	[88]
Polystyrene resins on oleic acid–Fe_3_O_4_	Co-precipitation	Not reported	Cd(II)	q_m_ = 88.56 mg/g	[89]
Polystyrene–divinylbenzene–Cyanex272–Fe_3_O_4_	Co-precipitation	M_s_ = 3.2 emu/g	Cd(II)	q_m_ = 17.77 mg/g	[90]
Humic acid/L-cystein–Fe_3_O_4_	Co-precipitation	Not reported	Hg(II)	q_m_ = 206.5 mg/g	[91]
Holloysite nanotube–Fe_3_O_4_	Co-precipitation	M_s_ = 27.91 emu/g	Methylene blue Neutral red Methyl orange	q_m_ = 18.44 mg/g q_m_ = 13.62 mg/g q_m_ = 0.65 mg/g	[92]
Ce-MOF modified activated carbon–Fe_3_O_4_	Co-precipitation	M_s_ = 21.39 emu/g	Methylene blue Indigo carmine	Removal efficiency =98–99% Removal efficiency =98–99%	[93]
N-vinylpyrrolidon/chitosan nanocomposite hydrogel–Fe_3_O_4_	Co-precipitation	M_s_ = 12 emu/g	Methyl orange	q_m_ ≈ 750 mg/g	[94]
β-cyclodextrin grafted carbon nanotube–Fe_3_O_4_	Co-precipitation	M_s_ = 7.15 emu/g	Methylene blue	q_m_ = 196.5 mg/g	[95]
Activated carbon– Fe_3_O_4_/γ-Fe_2_O_3_	Solvothermal	M_s_ > 30 emu/g	Methylene blue	q_m_ = 196.5 mg/g	[96]
Mineral derived silica–Fe_2_O_3_ and plant derived silica–Fe_2_O_3_	Precipitation, impregnation, and calcination	M_s_ ≈ 0.5–1.3 emu/g	Methylene blue	q_m_ = 7.0–27.3 mg/g	[97]
Activated sericite clay–Fe_3_O_4_	Co-precipitation	M_s_ = 2.17–8.12 emu/g	Methylene blue Crystal violet	Removal efficiency =99% Removal efficiency =99%	[98]
Poly(itaconic acid)/Fe_3_O_4_–sepiolite	Co-precipitation	M_s_ = 21.78 emu/g	Methylene blue Methyl violet	q_m_ = 196.08 mg/g q_m_ = 175.44 mg/g	[99]
Bentonite/APTMA–Fe_3_O_4_	Co-precipitation	M_s_ = 0.7 emu/g	Crystal violet Congo red	q_m_ = 2286 mg/g q_m_ = 1210 mg/g	[100]
MoS_2_@bentonite–Fe_3_O_4_	Co-precipitation	M_s_ = 11.448 emu/g	Crystal violet	q_m_ = 384.61 mg/g	[101]
Activated carbon–γFe_2_O_3_/Fe_3_O_4_/α-FeOOH	Co-precipitation	M_s_ = 38.5 emu/g	Malachite green	q_m_ = 486 mg/g	[102]
Lignosulfonate–Fe_3_O_4_	Co-precipitation	M_s_ = 43.98 emu/g	Rhodamine B	q_m_ = 22.47 mg/g	[84]
Reduced graphene oxide–Fe_3_O_4_	Co-precipitation	M_s_ = 51.76 emu/g	Rhodamine B	q_m_ = 432.91 mg/g	[103]
Mesoporous carbon–Fe_3_O_4_	Solvothermal	M_s_ = 28.89 emu/g	Ciprofloxacin	q_m_ = 98.28 mg/g	[104]
Polyacrylonitrile–Fe_3_O_4_	Solvothermal	Not reported	Tetracycline	q_m_ = 257.07 mg/g	[105]
Polypyrrole–chitosan–Fe_3_O_4_	Co-precipitation	M_s_ = 22.30 emu/g	Carbamazepine	q_m_ = 121.95 mg/g	[106]
Graphene oxide/cyclodextrin composite–Fe_3_O_4_	Solvothermal	M_s_ = 43.96 emu/g	Brivaracetam Eslicarbazepine acetate Carbamazepine	q_m_ = 36.38 mg/g q_m_ = 106.86 mg/g q_m_ = 54.49 mg/g	[107]
Carbon nanofiber aerogels–Fe/Fe_3_O_4_ core-shell	Co-precipitation	M_s_ = 102 emu/g	Oil and organic solvents	q_m_ = 37,000–87,000 mg/g	[108]
**Synthesis of adsorbents on magnetic particles**					
La(OH)_3_–Fe_3_O_4_	Precipitation	M_s_ = 15–20 emu/g	Phosphate	q_m_ = 11.77 mg/g	[109]
C18-functionalized silica–Fe_3_O_4_	Sol–gel	M_s_ = 39.19 emu/g	Phosphate	q_m_ = 0.3143 mg/g	[12]
P zeolite–Fe_3_O_4_	Hydrothermal	M_s_ = 2.8855 emu/g	K(I)	q_m_ = 215.1 mg/g	[110]
Mordenite zeolite–Fe_3_O_4_	Hydrothermal	Magnetic collection rate = 95%	Cs(I)	Removal efficiency >95%	[111]
Amino functionalized silica–Fe_3_O_4_	Sol–gel	M_s_ = 60.6 emu/g	Cr(III) Cr(VI)	q_m_ = 8.22 mg/g q_m_ = 11.4 mg/g	[112]
Poly(m-phenylenediamine)–Fe_3_O_4_	Oxidation-polymerization	M_s_ = 73.78–127.33 emu/g	Cr(VI)	q_m_ = 125.62–246.09 mg/g	[113]
SDS-PAN functionalized alumina–Fe_3_O_4_	Direct precipitation	Not reported	Co(II)	Recovery =95.6–98.8%	[114]
ZSM-5 zeolite–Fe_3_O_4_	Hydrothermal	M_s_ = 0.8743 emu/g	Pb(II)	q_m_ = 176.76 mg/g	[115]
Graphene oxide–LDH–Fe_3_O_4_	Milling and hydrothermal	M_s_ = 3.5 emu/g	Pb(II)	q_m_ = 39.7 mg/g	[116]
Amino functionalized silica–Fe_3_O_4_	Sol–gel	M_s_ = 29.3 emu/g	Pb(II)	q_m_ = 238 mg/g	[117]
NaA zeolite–Fe_3_O_4_	Hydrothermal	χ_ρ_ = 225–515 m^3^/kg	Cu(II) Pb(II)	q_m_ = 146 mg/g q_m_ = 477 mg/g	[118]
ZIF-8–Fe_3_O_4_	Stirring	M_s_ = 37.26 emu/g	Pb(II) Cu(II)	q_m_ = 719.42 mg/g q_m_ = 301.33 mg/g	[119]
Carboxymethylated lignin functionalized silica–Fe_3_O_4_	Sol–gel	Not reported	Pb(II) Cu(II)	q_m_ = 150.33 mg/g q_m_ = 70.69 mg/g	[120]
MnO_2_–Fe_3_O_4_	Hydrothermal	M_s_ = 14.19 emu/g	Cu(II) Cd(II) Zn(II) Pb(II)	q_m_ = 498.575 mg/g q_m_ = 439 mg/g q_m_ = 416.5 mg/g q_m_ = 490.5 mg/g	[27]
Siloxydithiocarbamate functionalized silica–Fe_3_O_4_	Sol–gel	M_s_ ≈ 70 emu/g	Hg(II)	Removal efficiency > 99.8%	[121]
DPTH-functionalized silica–Fe_3_O_4_	Sol–gel	Not reported	Hg(II)	q_m_ = 8.39 mg/g	[122]
Sulfur functionalized amide linked organic polymer–MNP-NH_2_	Sol–gel	M_s_ = 15 emu/g	Hg(II)	q_m_ = 512 mg/g	[123]
Microbial extracellular polymeric substances coated Fe_3_O_4_	Oxidative copolymerization	M_s_ = 79.01 emu/g	Ag(I)	q_m_ = 48 mg/g	[124]
Hydrothermal carbon modified with NaOH–Fe_3_O_4_	Hydrothermal	Not reported	U(VI)	q_m_ = 761.20 mg/g	[125]
Amidoxime functionalized flower-like TiO_2_ microspheres–Fe_3_O_4_	Sol–gel	M_s_ = 15.19 emu/g	U(VI)	q_m_ = 313.6 mg/g	[126]
Amino-methylene-phosphonic-functionalized silica–Fe_3_O_4_	Sol–gel	Not reported	Sb(III)	q_m_ ≈ 130 mg/g	[127]
Thiol functionalized silica–Fe_3_O_4_	Sol–gel	Not reported	[AuCl4]^−^	q_m_ = 115 mg/g	[128]
C18-Silica–Fe_3_O_4_	Sol–gel	M_s_ = 41.31 emu/g	Sudan dyes	Recovery = 91–104%	[129]
TiO_2_/HKUST-1–Fe_3_O_4_	Spray-assisted synthesis	M_s_ = 1.62 emu/g	Methylene blue	q_m_ > 700 mg/g	[130]
ZIF-8–Fe_3_O_4_	Mixing and heating	M_s_ = 14.38 emu/g	Methylene blue	q_m_ = 20.2 mg/g	[131]
Poly(propylene imine)-functionalized UiO-66–Fe_3_O_4_	Solvothermal	M_s_ = 10.5 emu/g	Acid blue 92 Direct red 31	q_m_ = 122.5 mg/g q_m_ = 173.7 mg/g	[132]
Chitosan-based adsorbent modified with AO–Fe_3_O_4_	Sol-gel	M_s_ = 12.03 emu/g	Orange II Acid red 88 Red amaranth	q_m_ = 955.0 mg/g q_m_ = 1075.8 mg/g q_m_ = 567.5 mg/g	[133]
Polydopamine-coated Fe_3_O_4_ modified with deep eutectic solvents	Self-polymerization	M_s_ = 65.71 emu/g	Malachite green Sunset yellow FCF	q_m_ = 277.78 mg/g q_m_ = 129.27 mg/g	[134]
Sulfamic acid-functionalized polyamidoamine–Fe_3_O_4_	Ultrasonication	M_s_ = 25 emu/g	Malachite green	q_m_ = 1250 mg/g	[135]
Sulfonic acid functionalized covalent organic polymer–Fe_3_O_4_	Sol–gel	M_s_ = 20.2 emu/g	Malachite green	q_m_ = 333.4 mg/g	[136]
Cationic surfactant functionalized silica–Fe_3_O_4_	Sol–gel	Not reported	Metal ion-8-hydroxyquinoline complexes	Recovery = 93–113%	[137]
C18-functionalized Fe_3_O_4_ caged in Ba^2+^-alginate	Solvothermal	M_s_ = 49.31 emu/g	PAHs Phthalate esters	Recovery = 72–108%	[138]
C18-modified interior pore wall mesoporous silica–Fe_3_O_4_	Sol–gel	M_s_ = 40.8 emu/g	Phthalates	Not reported	[139]
Graphene oxide–LDH–Fe_3_O_4_	Milling and hydrothermal	M_s_ = 3.5 emu/g	2,4-dichlorophenoxyacetic acid	q_m_ = 173 mg/g	[116]
Agarose coated silica modified with SDS–Fe_3_O_4_	Sol–gel	M_s_ = 21.57 emu/g	Phenazopyridine monohydrochloride	q_m_ = 41 mg/g	[140]
Covalent organic framework–Fe_3_O_4_	Sol–gel	M_s_ = 15.8 emu/g	Diclofenac sodium	q_m_ = 565 mg/g	[141]

M_s_: saturation magnetization; χ_ρ_ = magnetic susceptibility; q_m_: maximum adsorption capacity; AO: acryloyloxyethyl dimethylbenzyl ammonium chloride; APTMA: 3-acrylamidopropyltrimethylammonium chloride; CSSNa: sodium dithiocarbamate; DTPA: diethylenetriamine pentaacetic acid; HKUST-1: Cu_3_(1,3,5-benzenetricarboxylate)_2_; LDH: layered double hydroxide; MNP-NH_2_: Fe_3_O_4_@SiO_2_−NH_2_; MOF: metal organic framework; PAHs: polyaromatic hydrocarbons; PAN: 1-(2-pyridylazo)-2-naphthol; PGMA: polyglycidyl methacrylate; SDS: sodium dodecylsulfate; ZIF-8: Zn(2-methylimidazole)_2_.

**Table 2:** Advanced synthesis methods of magnetic adsorbents, and their performance on adsorption of organic and inorganic pollutants in water.

Adsorbents	Synthesis Method	Magnetic Properties	Pollutant(s)	Adsorption or Removal Performance	Reference
**Advanced synthesis of magnetic materials as adsorbents**					
Sulfur-doped Fe_3_O_4_	Simple mixing and sintering	M_s_ = 37.1 emu/g	As(V)	q_m_ = 58.38 mg/g	[48]
Dendrimerlike biosorbent–Fe_3_O_4_/Fe_2_O_3_ based on orange peel waste	Co-precipitation	Not reported	As(V)	q_m_ = 81.3 mg/g	[173]
Sulfur-doped Fe_3_O_4_	Simple mixing and sintering	M_s_ = 32.97 emu/g	Pb(II)	q_m_ = 500 mg/g	[47]
β-cyclodextrin-stabilized Fe_3_S_4_	Thermal decomposition	M_s_ = 37.1 emu/g	Pb(II)	q_m_ = 256.41 mg/g	[174]
Fe_3_S_4_-reduced graphene oxide	Thermal decomposition and sulfuration	M_s_ = 20.67 emu/g	Pb(II)	q_m_ = 285.71 mg/g	[49]
Fe/FeS	Sulfidation	M_s_ = 78.0 emu/g	Cr(VI)	q_m_ = 69.7 mg/g	[175]
MgFe_2_O_4_	Sol–gel	M_s_ = 9.4 emu/g	Indigo carmine dye	q_m_ = 46 mg/g	[176]
CuFe_2_O_4_	Solution combustion	M_s_ = 18.1 emu/g	Malachite green	q_m_ = 22 mg/g	[177]
Bio-synthesized Fe_3_O_4_	Simple precipitation using microalgae extract	M_s_ = 0.2705 emu/g	Crystal violet Methyl orange	q_m_ = 256.41 mg/g q_m_ = 270.27 mg/g	[21]
Bio-synthesized Fe_3_O_4_	Simple precipitation using microalgae extract	M_s_ = 0.2705 emu/g	Methylene blue	q_m_ = 312.5 mg/g	[22]
Starch-coated Fe_3_O_4_	Green co-precipitation	M_s_ = 46.8 emu/g	Optilan blue	Removal efficiency =72–89%	[178]
S-nZVI	Sulfidation	Not reported	Florfenicol	Removal efficiency >98%	[179]
S-nZVI	Sulfidation	Not reported	Diclofenac	Removal efficiency >85.9%	[180]
**Advanced synthesis of magnetic particles on adsorbents**					
Pinewood-derived biochar–MnFe_2_O_4_	Direct pyrolysis	Not reported	As(V)	q_m_ = 3.44 g/kg	[181]
Pinewood-derived biochar–γ-Fe_2_O_3_	Direct pyrolysis	Not reported	As(V)	q_m_ = 428.7 mg/kg	[182]
Sodium alginate-dispersed nZVI	Sulfidation	Not reported	Cr(VI)	Removal efficiency =96.4%	[183]
Fe-coated bamboo charcoal	Impregnation and microwave heating	Not reported	Pb(II)	q_m_ = 200.38 mg/g	[184]
Bagasse-derived biochar	Co-precipitation	M_s_ = 0.49–1.17 emu/g	17β-estradiol	q_m_ = 34.06–50.24 mg/g	[185]
Biotemplate-fabricated ZnFe_2_O_4_/MgAl LDH	Thermal decomposition	M_s_ = 31.8 emu/g	Congo red	q_m_ = 294.12 mg/g	[186]
Ag–C–Fe_3_O_4_	Solution combustion	M_s_ = 2.6 emu/g	Methylene blue Acid orange 7 Rhodamine 6G	q_m_ = 152.62 mg/g q_m_ = 154.57 mg/g q_m_ = 168.68 mg/g	[187]
Activated carbon–Fe_3_O_4_	Solution combustion	M_s_ = 4.82–13.5 emu/g	Acid yellow 42 Acid red 213	q_m_ = 62.36 mg/g q_m_ = 77.99 mg/g	[188]
C–Fe_3_O_4_	Solution combustion	M_s_ = 2.43 emu/g	Acid orange 7 Acid blue 129 Methylene blue Rhodamine 6G	q_m_ = 126.19 mg/g q_m_ = 83.42 mg/g q_m_ = 118.15 mg/g q_m_ = 131.80 mg/g	[189]
**Advanced synthesis of adsorbents on magnetic particles**					
Faujasite-type zeolite–Fe_3_O_4_	Seed-assisted hydrothermal with seed crystal/Fe_3_O_4_ mixture	Not reported	Methylene blue	q_m_ = 35.7 mg/g	[29]
Activated carbon–Fe_3_O_4_	Carbonization of Fe_3_O_4_ embedded polymer precursor	Not reported	Methylene blue	q_m_ = 650 mg/g	[34]
BEA-type zeolite–Fe_3_O_4_	Dry-gel conversion of Fe_3_O_4_ pre-mixed precursor gel	Not reported	Methylene blue	q_m_ = 133 mg/g	[31]
Zn-based zeolitic Imidazolate MOF-basil seed mucilage nanocomposite	Ultrasonication	M_s_ = 2.22 emu/g	Methylene blue Eriochrome black T	q_m_ = 9.09 mg/g q_m_ = 13.21 mg/g	[190]
MOR-type zeolite–Fe_3_O_4_	Seed-assisted hydrothermal with seed crystal/Fe_3_O_4_ mixture	Not reported	Benzene	q_m_ = 6.9 mg/g	[30]
**Co-synthesis of magnetic particles and adsorbents**					
ZrO_2_–Fe_3_O_4_	Co-precipitation	M_s_ > 23.65 emu/g	Phosphate	q_m_ = 27.93–69.44 mg/g	[26]
Ma/Al/La–Fe_3_O_4_	Co-precipitation and calcination	Not reported	F^−^	q_m_ = 65.75 mg/g	[191]
Triethylene tetramine functionalized chitosan resin–Fe_3_O_4_	Precipitation and crosslinking	M_s_ = 30 emu/g	Uranyl	q_m_ = 166.6 mg/g	[192]
MgAl LDH on carbon–Fe_3_O_4_	Hydrothermal self-assembly and Sol–gel	M_s_ = 5.84 emu/g	Cr(VI)	q_m_ = 152.0 mg/g	[193]
Rice husk-derived carbonaceous material–Fe_3_O_4_	Carbon-thermal	M_s_ = 77.8 emu/g	Cr(VI)	q_m_ = 157.7 mg/g	[194]
Sludge biochar–Fe_3_O_4_	Hydrothermal	M_s_ = 29.94 emu/g	Pb(II)	q_m_ = 174.216 mg/g	[195]
Biochar–Fe_3_O_4_	Electromagnetization and pyrolysis	M_s_ = 26.79 emu/g	Acid orange 7	q_m_ = 382.01 mg/g	[33]
Fullerene–Fe_3_O_4_	Solvent-free catalytic thermal decomposition	M_s_ = 7.002 emu/g	Acid blue 25 Methylene blue	q_m_ = 806.5 mg/g q_m_ = 833.3 mg/g	[196]
Polyvinylpyrrolidone–Fe_3_O_4_	Modified hydrothermal	Not reported	Crude oil	Removal efficiency ≈100%	[197]

M_s_: saturation magnetization; q_m_: maximum adsorption capacity; LDH: layered double hydroxide; MOF: metal organic framework; S-nZVI: sulfidized nano zerovalent iron.

## Data Availability

No new data were created or analyzed in this study. Data sharing is not applicable.
